# ReduXis: A Comprehensive Framework for Robust Event-Based Modeling and Profiling of High-Dimensional Biomedical Data

**DOI:** 10.3390/ijms26188973

**Published:** 2025-09-15

**Authors:** Neel D. Sarkar, Raghav Tandon, James J. Lah, Cassie S. Mitchell

**Affiliations:** 1 Department of Biomedical Engineering, Georgia Institute of Technology, Atlanta, GA 30332, USA; nsarkar7@gatech.edu (N.D.S.);; 2 Center for Machine Learning, Georgia Institute of Technology, Atlanta, GA 30332, USA; 3 Department of Neurology, Emory School of Medicine, Atlanta, GA 30307, USA; jlah@emory.edu

**Keywords:** event-based modeling, biomarker discovery, disease progression, omics-driven profiling, multimodal data integration, machine learning, artificial intelligence, colorectal adenocarcinoma, transitional cell carcinoma, Alzheimer’s disease

## Abstract

Event-based models (EBMs) are powerful tools for inferring probabilistic sequences of monotonic biomarker changes in progressive diseases, but their use is often hindered by data quality issues, high dimensionality, and limited interpretability. We introduce ReduXis, a streamlined pipeline that overcomes these challenges via three key innovations. First, upon dataset upload, ReduXis performs an automated data readiness assessment—verifying file formats, metadata completeness, column consistency, and measurement compatibility—while flagging preprocessing errors, such as improper scaling, and offering actionable feedback. Second, to prevent overfitting in high-dimensional spaces, ReduXis implements an ensemble voting-based feature selection strategy, combining gradient boosting, logistic regression, and random forest classifiers to identify a robust subset of biomarkers. Third, the pipeline generates interpretable outputs—subject-level staging and subtype assignments, comparative biomarker profiles across disease stages, and classification performance visualizations—facilitating transparency and downstream analysis. We validate ReduXis on three diverse cohorts: the Emory Healthy Brain Study (EHBS) cohort of patients with Alzheimer’s disease (AD), a Genomic Data Commons (GDC) cohort of transitional cell carcinoma (TCC) patients, and a GDC cohort of colorectal adenocarcinoma (CRAC) patients.

## 1. Introduction

Chronic, progressive diseases—ranging from neurodegenerative disorders such as Alzheimer’s disease (AD), Parkinson’s disease, and frontotemporal dementia to malignancies including breast, colorectal, and lung cancer—represent some of the most formidable challenges in modern medicine. Their burden stems not only from their prevalence and lack of curative therapies but also from their insidious onset, substantial heterogeneity, and complex biological underpinnings. In neurodegenerative diseases, hallmark pathological changes—such as β-amyloid deposition, tau-mediated neurofibrillary tangles, and region-specific cortical atrophy—often begin years or even decades prior to the onset of clinical symptoms [1,2,3]. Similarly, oncogenesis proceeds through a cascade of molecular and microenvironmental events, including somatic mutations, immune evasion, stromal remodeling, and angiogenesis [4].

Although these diseases differ in their underlying pathophysiology, they share a key characteristic: progression is marked by a sequential, often monotonic, accumulation of molecular and cellular alterations. Accurately characterizing the temporal sequence of these biomarker transitions is critical for early detection, prognostic stratification, and the development of stage-specific interventions [5]. However, comprehensive longitudinal datasets that capture the full course of disease progression are uncommon, and cross-sectional cohorts inherently lack temporal information.

Event-based models (EBMs) address this limitation by providing a probabilistic framework for reconstructing the most likely sequence of biomarker changes from cross-sectional data. These models perform a form of soft clustering, assigning individuals a probabilistic disease stage based on their biomarker profile and the inferred progression sequence. EBMs underpin frameworks such as Subtype and Stage Inference (SuStaIn) [6], which have been applied successfully across diverse diseases and data modalities to elucidate both temporal and phenotypic heterogeneity in disease progression.

Recent extensions of EBMs—including variants by Young et al. [6] and Wijeratne et al. [5]—have further advanced the field by introducing mechanisms for modeling subtype variability, incorporating multimodal data, and improving statistical efficiency. However, these approaches often assume well-curated datasets with minimal preprocessing inconsistencies, and they may require extensive tuning or domain-specific customization. Additionally, interpretability remains limited in many existing pipelines, with outputs that are often difficult for non-expert users to translate into actionable biological or clinical insights.

Open-source tools such as pySuStaIn [7] and s-SuStaIn [8] have significantly increased the accessibility of event-based modeling (EBM) methodologies, facilitating their adoption across diverse biomedical domains. For instance, in Alzheimer’s disease research, these tools have been applied to cerebrospinal fluid (CSF) proteomics to reconstruct disease progression trajectories using cross-sectional data. Notably, Tandon et al. [9] implemented s-SuStaIn on the ADNI dataset—a widely used cohort in AD research—to identify biomarker-based subtypes and stages in Alzheimer’s progression. Similar applications to multimodal neuroimaging have also been demonstrated in frontotemporal dementia [10]. Nonetheless, widespread adoption of EBMs in translational biomedical research remains limited due to three critical barriers. First, the heterogeneity of biomedical datasets—characterized by inconsistent formats, missing metadata, and preprocessing errors—poses a major obstacle to standardized analysis. Second, the high dimensionality of clinical and omics data increases the risk of model overfitting and instability in inferred biomarker sequences. Third, current EBM pipelines often produce outputs that are challenging to interpret, lacking intuitive visualizations and clinical usability.

To overcome these limitations, we introduce ReduXis, a comprehensive, user-friendly EBM pipeline tailored for translational research applications. ReduXis begins with automated data validation, ensuring appropriate formatting, metadata integrity, and preprocessing consistency, and offers immediate feedback to resolve issues prior to analysis. To enhance modeling stability, ReduXis incorporates a robust ensemble-based feature selection strategy, using a majority-voting scheme across gradient boosting, logistic regression, and random forest classifiers [7,11,12]. Finally, the pipeline generates a rich set of interpretable results, including subject-level subtype and stage assignments, biomarker trajectories across disease stages, and interactive performance metrics.

By integrating rigorous data readiness assessments, stable feature selection, and transparent reporting into a unified interface, ReduXis offers a practical, reproducible, and interpretable approach to event-based modeling. Unlike prior EBM extensions that emphasize methodological complexity or theoretical performance, ReduXis is designed explicitly for real-world biomedical data — prioritizing robustness, usability, and clarity. This positions it as a valuable tool for researchers aiming to uncover temporal biomarker signatures and heterogeneity in complex, progressive diseases.

## 2. Results and Discussion

ReduXis was designed to enable stage and subtype event-based models (EBMs) to be applied to very large data sets, including high-dimensional omics data, with relative ease and speed. To evaluate the generalizability, interpretability, and robustness of the developed ReduXis pipeline and corresponding Python package, we applied it to three distinct case studies spanning neurodegeneration and oncology. These datasets were selected to represent varying levels of complexity in outcome structure, dimensionality, and the need for subtyping. In contrast to conventional pipelines, which may fail silently or halt on malformed inputs, ReduXis completed all runs without error across at least 12 independent executions per dataset. The high consistency of outputs—including identical event sequences across repeated trials—demonstrates regularity and strong reproducibility, two critical properties for translational biomedical applications. Together, these results strongly support that ReduXis enables robust, generalizable, and interpretable event-based modeling across very large, heterogeneous disease datasets.

### 2.1. Case Study 1: Colorectal Adenocarcinoma

Colorectal adenocarcinoma (CRAC) is the most common histological subtype of colorectal cancer, arising from the epithelial cells lining the colon or rectum. Globally, CRAC ranks as the third to fourth most frequently diagnosed cancer and is a leading cause of cancer-related mortality, with over 900,000 deaths annually [13,14]. Despite advances in screening and treatment, challenges persist due to its molecular heterogeneity, late-stage diagnosis in many patients, and variable response to standard therapies [13]. Early detection and accurate molecular staging are, therefore, critical for improving prognosis and guiding personalized interventions. In this context, event-based models can uncover the temporal ordering of oncogenic and tumor-suppressor gene alterations, offering insights into CRAC progression that complement existing histopathological and clinical assessments.

To illustrate the utility of ReduXis in this context, we applied it to a publicly available RNA-seq dataset of colorectal adenocarcinoma (CRAC) patients from the Genomic Data Commons, specifically utilizing the TCGA-COAD project. This dataset was selected as a baseline use case due to its binary classification structure (“Normal” vs. “Tumor”) and the absence of confounding molecular subtypes, enabling clearer interpretation of biomarker progression patterns. The original gene expression matrix, comprising approximately 5100 genes, was reduced to 66 high-importance features using our ensemble-based stability selection procedure, as detailed in Section 3. These selected features were then used to construct an event-based model of disease progression. Notably, several of the top-ranked genes identified by ReduXis (e.g., *TRIB3*, *GSN*) have well-established roles in regulating cell survival, cell cycle deregulation, and apoptosis evasion. A full summary of key genes implicated in CRAC, including gene-level functions and literature-supported biological relevance, is provided in Table A1.

**Figure 1:**
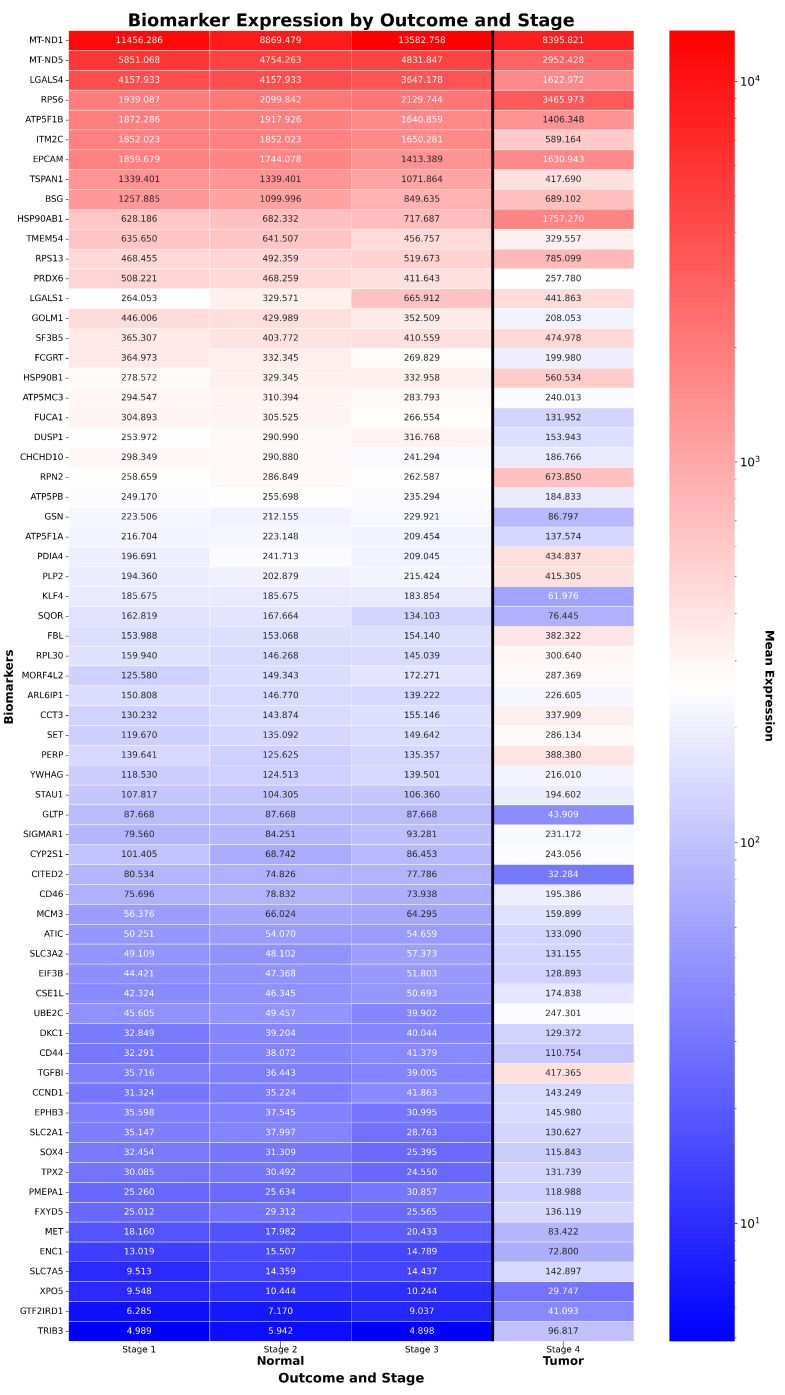
*Expression of CRAC biomarkers across stages and outcomes.* Heatmap of 66 ReduXis-prioritized biomarkers for CRAC staging, ordered by average expression in normal tissue. ReduXis applied value clipping and rescaling at [Q3 + 1.5 × IQR] to minimize the outsized influence of extreme-expression features, ensuring stage assignment reflected the collective pattern across all 100 biomarkers—each still weighted by individual staging impact. Expression levels are color-mapped (red = high, blue = low), revealing increased heterogeneity in tumor samples, especially at later stages. Notably, expression dynamics of canonical CRAC drivers are captured, including upregulated oncogenes *RPN2* and *TRIB3*, and downregulated tumor suppressors *GSN* and *KLF4*.

**Figure 2:**
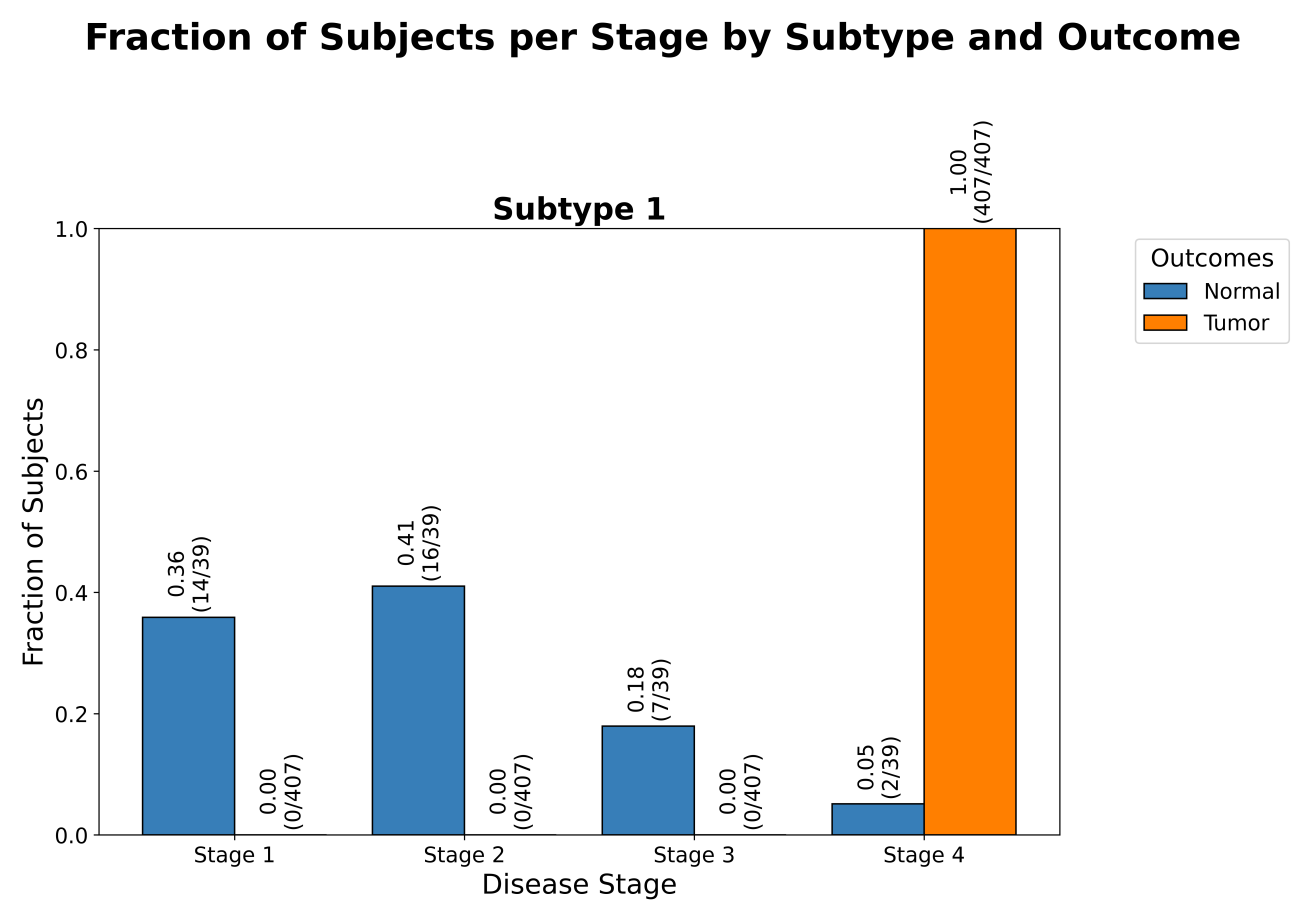
*Stage distribution by outcome in CRAC.* Grouped bar chart of stage assignments shows that normal subjects cluster predominantly in Stages 1 and 2, while tumor samples span Stages 3 and 4. Stage assignments reflect biologically coherent transitions from normal to advanced tumor states, with a statistically significant (p<0.001) degree of separation between the Normal and Tumor outcomes, as determined by a $\chi^{2}$ goodness of fit test, further detailed in Table A2.

**Figure 3:**
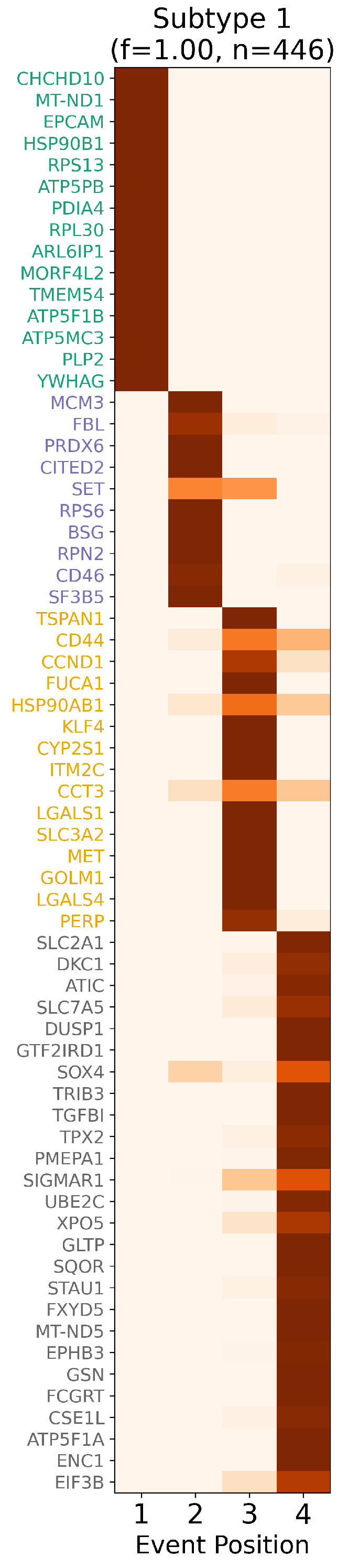
*Inferred staging order of CRAC biomarkers.* Event-based progression matrix illustrating ReduXis’ confidence in assigning each biomarker to a specific colorectal adenocarcinoma (CRAC) disease stage. Color intensity (shades of red) denotes staging confidence, with darker hues representing higher certainty. Notably, the majority of biomarkers exhibit deep red tones, highlighting ReduXis’ robustness even after dimensionality reduction—from approximately 5100 initial features to a distilled set of 66—without sacrificing staging precision. The visibly uneven cluster sizes and slight uncertainty of inferred disease stages are biologically expected, reflecting CRAC’s inherent molecular complexity: a multifaceted cascade involving genomic instability, epigenetic alterations, dysregulated signaling pathways, and tumor-immune microenvironment dynamics.

As shown in Figure 1, Figure 2 and Figure 3, ReduXis identified *TRIB3*, *RPN3*, *GSN*, and *KLF4* as differentially expressed and biologically relevant to CRAC progression. Importantly, these genes contributed to a distinctive pattern wherein normal tissue samples predominantly aligned with earlier progression stages, while tumor tissue samples were almost exclusively associated with later stages. Given that all four genes were inferred to belong to stages beyond Stage 1, and that Figure 2 shows all tumor samples localized to Stage 4, this suggests their potential significance in the transition from normal to precancerous to malignant states.

### 2.2. Case Study 2: Transitional Cell Carcinoma

**Figure 4:**
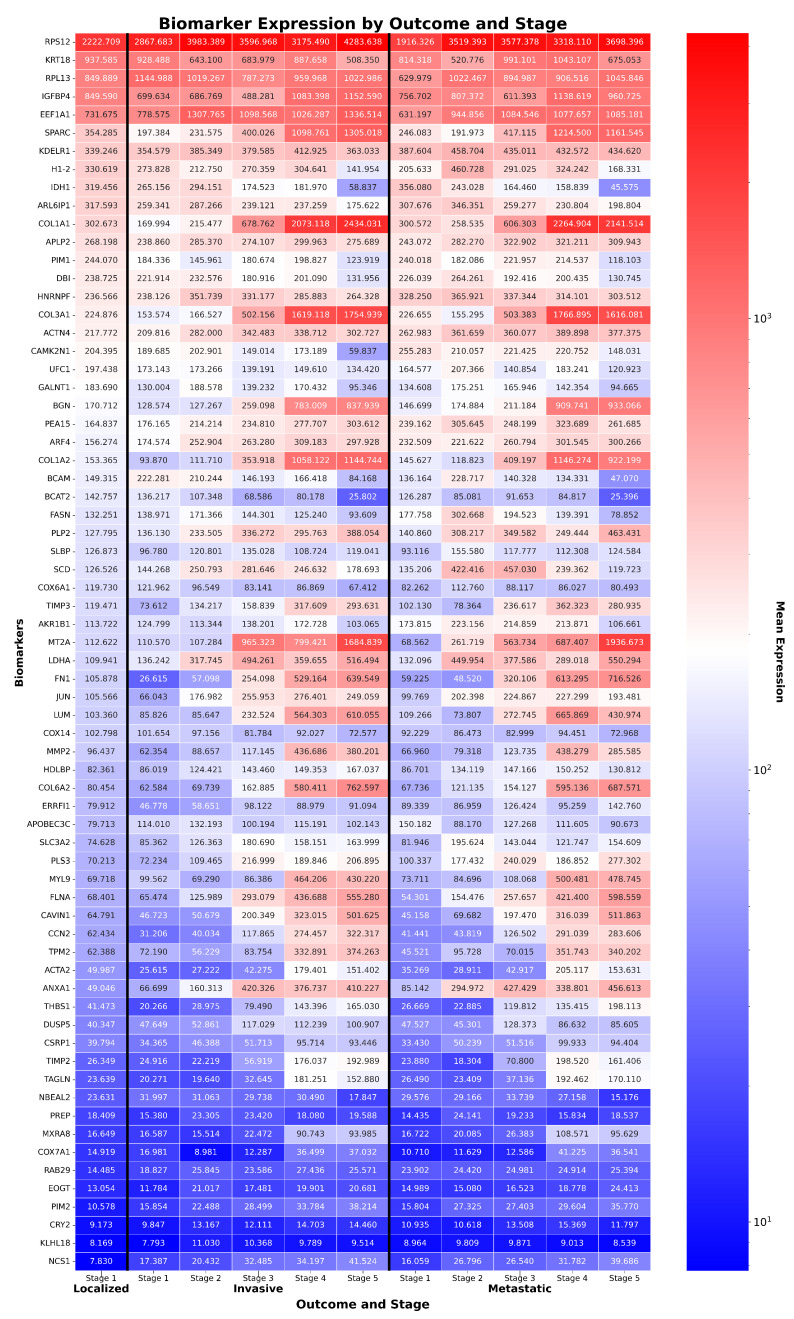
*Expression of TCC biomarkers across stages and outcomes.* Heatmap of 68 ReduXis-prioritized biomarkers for transitional cell carcinoma (TCC) staging, selected independently from those used in CRAC to reflect ReduXis’s dataset-specific biomarker discovery approach. Expression values were clipped and rescaled using [Q3 + 1.5 × IQR] thresholds to mitigate the influence of extreme-expression outliers. This preserves meaningful variability across features while ensuring that stage inference is informed by all 68 biomarkers—not disproportionately driven by a few—with relative effect sizes maintained rather than overly standardized. Color scale represents expression (red = high, blue = low), with tumor samples showing distinct expression divergence across stages. Functionally important biomarkers include tumor suppressors *IDH1* and *H1-2*, and oncogenes *COL3A1* and *MXRA8*.

**Figure 5:**
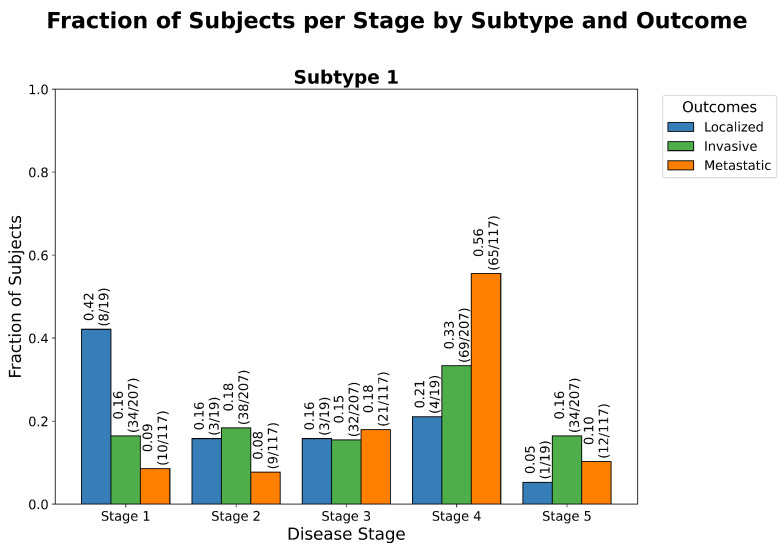
*Stage distribution by outcome in TCC.* Grouped bar chart showing the distribution of inferred disease stages across clinical outcome groups for transitional cell carcinoma (TCC): “Localized”, “Invasive”, and “Metastatic”. As expected, “Localized” samples are predominantly assigned to early stages, “Invasive” samples span a broader range of stages, and “Metastatic” samples cluster in the later stages—reflecting a biologically plausible trajectory of tumor progression. The non-random nature of these assignments is supported by a statistically significant $\chi^{2}$ goodness-of-fit test (p<0.001), detailed in Table A4. This test evaluates whether the observed distribution of stage assignments deviates from what would be expected under a null hypothesis of random assignment (i.e., assuming uniform probability across outcomes regardless of biological data). The strong deviation from randomness indicates that ReduXis’s inferred stage assignments are meaningfully grounded in the biomarker expression data—not stochastic artifacts—capturing a coherent pattern of disease advancement across clinical groups.

**Figure 6:**
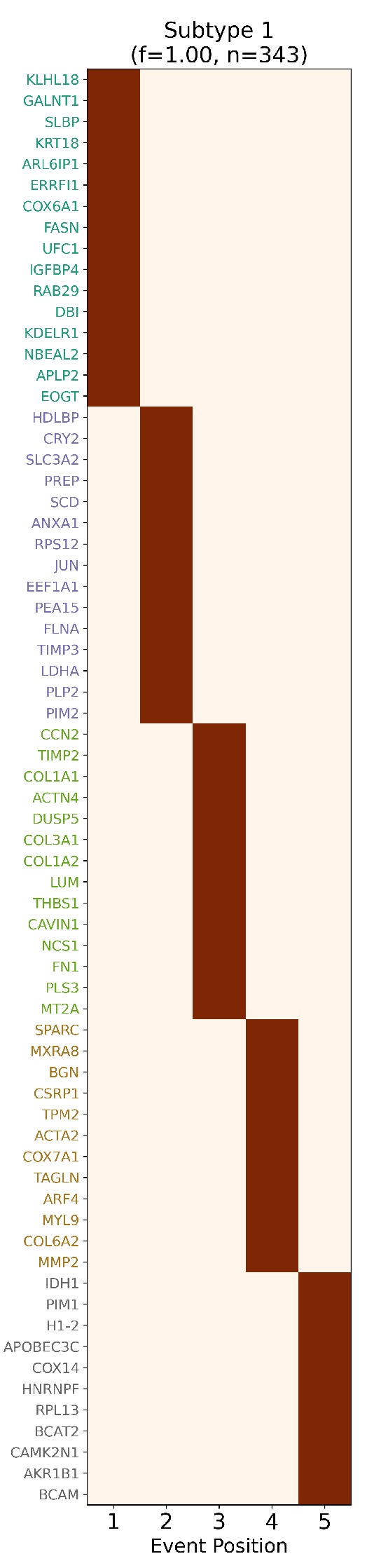
*Inferred staging order of TCC biomarkers.* Biomarker-based event progression matrix for transitional cell carcinoma (TCC), showing the inferred stage assignments of 68 ReduXis-prioritized biomarkers. Despite aggressive feature reduction—from over 5700 transcriptomic features down to just 68—ReduXis achieved high-confidence stage assignments, as reflected by the strong color intensity (deep maroon red = high confidence, light red = lower confidence). The model effectively leveraged distinct expression dynamics across biomarkers to anchor stage boundaries. This stagespecific marker distribution is consistent with canonical TCC biology, which features progressive hallmarks such as metabolic reprogramming (e.g., theWarburg effect), epithelial-to-mesenchymal transition (EMT) involving reduced adhesion and increased motility, compromised DNA repair, checkpoint bypass, and microenvironmental remodeling. The separation of biomarkers across stages further validates the temporal structure captured by ReduXis, demonstrating that meaningful biological transitions remain detectable even under extreme dimensionality reduction.

Bladder cancer is the tenth most common malignancy worldwide, with transitional cell carcinoma (TCC), also called urothelial carcinoma, accounting for approximately 90–95% of cases and contributing to over 200,000 deaths per year in the United States alone [15,16]. The disease’s high genomic heterogeneity, propensity for recurrence, and frequent late-stage diagnosis pose significant challenges for effective stratification and treatment [16]. Accurate molecular staging and intra-disease stratification remain critical unmet needs, as conventional clinicopathological criteria often fail to capture underlying biological diversity. To illustrate ReduXis’s utility in this context, we applied it to data derived from the publicly available TCGA-BLCA project under the TCGA program, which involves three clinical outcomes of TCC patients, “Localized” for low-grade, localized TCC, “Invasive” for medium-grade, muscle-invasive TCC, and finally “Metastatic” for high-grade, metastatic TCC. However, unlike in the TCGA-COAD dataset, these data had no healthy controls. Nevertheless, from an original RNA-seq matrix of approximately 5700 genes, ReduXis selected 68 high-importance biomarkers via ensemble stability scoring (see Materials and Methods), allowing us to test the framework’s ability to reveal distinct progression trajectories within a single disease entity. A full summary of key genes implicated in TCC, including gene-level functions and literature-supported biological relevance, is provided in Table A3. As shown in Figure 4, Figure 5 and Figure 6, ReduXis identified *COL3A1*, *MXRA8*, *IDH1*, and *H1-2* as differentially expressed and biologically relevant to TCC progression. Importantly, these genes contributed to a distinctive pattern wherein early-stage TCC samples predominantly aligned with earlier progression stages, while late-stage TCC tissue samples were associated with later stages, with significantly more overlap between outcomes as compared with the TCGA-COAD case study.

Given that the oncogenes *COL3A1* and *MXRA8* were inferred to contribute to progression through Stages 3 and 4, while the tumor suppressors *IDH1* and *H1-2* were implicated in Stage 5, our findings align well with the canonical model of cancer progression. This model typically involves early oncogenic events—such as upregulated signaling and increased metabolic activity—followed by later disruptions in tumor suppressor function, leading to unchecked cell cycle progression, genomic instability, invasion, and metastasis. The temporal stratification of gene involvement observed here supports this paradigm, with oncogene activation preceding tumor suppressor loss as the disease advances toward more aggressive stages.

### 2.3. Case Study 3: Emory Healthy Brain Study

The Emory Healthy Brain Study (EHBS) is a racially diverse, longitudinal cohort designed to facilitate biomarker discovery and disease trajectory modeling in Alzheimer’s disease (AD) through multimodal data integration. Participants, aged 50–75 years, were age-matched to individuals with clinically diagnosed AD and assessed via neuropsychological testing, PET imaging, and cerebrospinal fluid (CSF) proteomics [17,18]. Based on CSF tau:A $\beta_{42}$ ratios, individuals were stratified into three clinical groups: “Control” (biomarker-negative, cognitively normal; BM−/CN), “AsymAD” (biomarker-positive, cognitively normal; BM+/CN), and “AD” (biomarker-positive with clinical dementia; BM+/CD) [18]. Targeted proteomic analysis was performed on CSF samples from 392 EHBS participants, quantifying 71 peptides. Due to the dataset’s low dimensionality, no preliminary feature selection was required prior to applying ReduXis, a modeling framework optimized for subtype discovery and temporal staging.

To uncover the biological heterogeneity underlying AD, ReduXis identified three distinct molecular subtypes, consistent with previously reported metabolically rooted variants of AD [19]: (1) an inflammatory subtype, often associated with chronic systemic inflammation and infectious triggers; (2) a non-inflammatory subtype, linked more directly to canonical amyloid pathology and (3) a rare tau-dominant cortical atrophy subtype, characterized by widespread cortical involvement with relative hippocampal sparing. Using these subtypes, ReduXis reconstructed temporally resolved disease trajectories, enabling the identification of stage-specific CSF peptide expression patterns that precede or co-occur with cognitive decline.

Notably, an intriguing pattern emerged within the inflammatory AD subtype: multiple peptides linked to periodontal disease and oral dysbiosis—particularly those associated with the growth of *Porphyromonas gingivalis*—were enriched in this group (Table A5). This finding adds to the mounting body of evidence connecting chronic gum disease to neuroinflammation in Alzheimer’s disease, suggesting that peripheral immune challenges may modulate central nervous system vulnerability. These associations not only underscore the biological plausibility of the inferred subtypes, but also open compelling avenues for future exploration of the oral–systemic axis in neurodegeneration.

**Figure 7:**
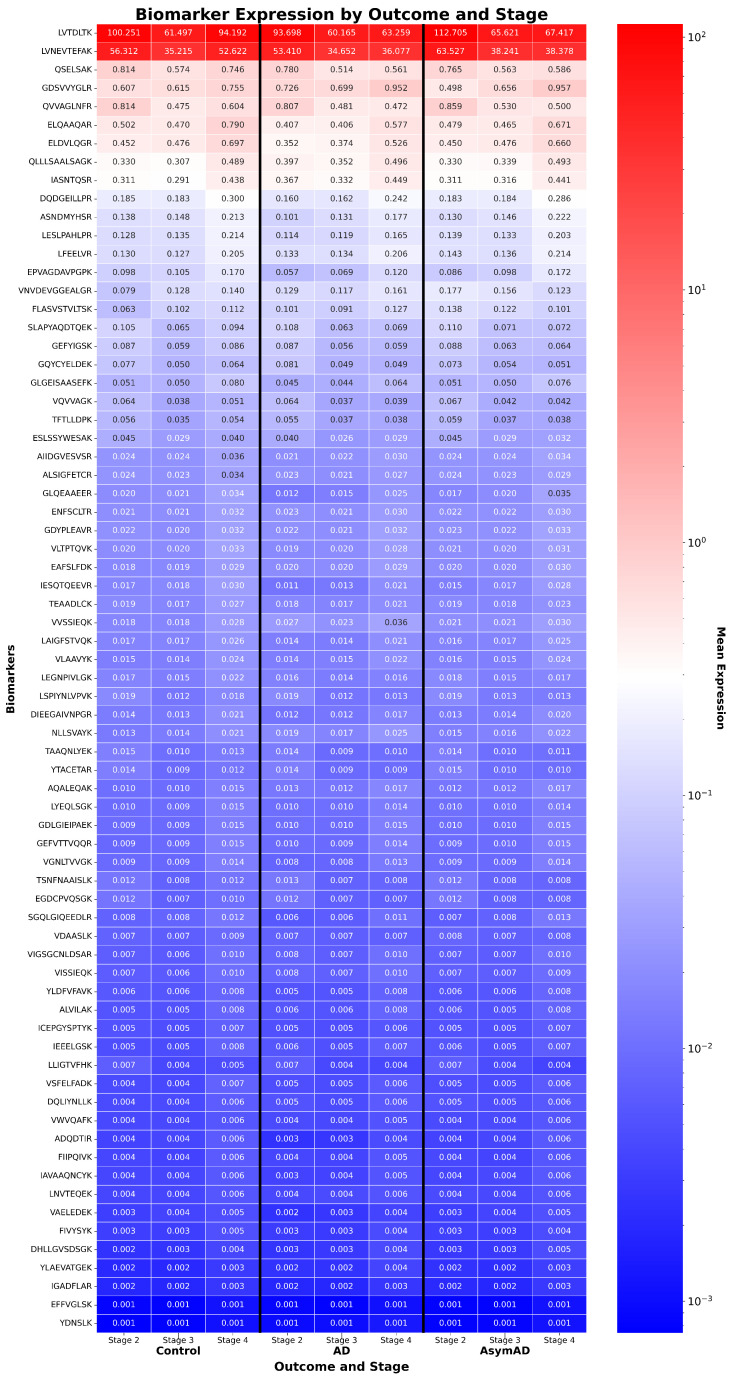
*CSF peptide expression by disease stage and outcome in EHBS.* Heatmap of 71 cerebrospinal fluid (CSF) peptides from the AD EHBS proteomics dataset, ordered by average expression in control subjects. Unlike prior transcriptomic datasets, no feature selection was necessary due to the lower dimensionality of the proteomic panel. Expression values were clipped and rescaled using the [Q3 + 1.5 × IQR] method to prevent a few highly expressed peptides from dominating stage inference, ensuring all 71 peptides contributed meaningfully to staging.

**Figure 8:**
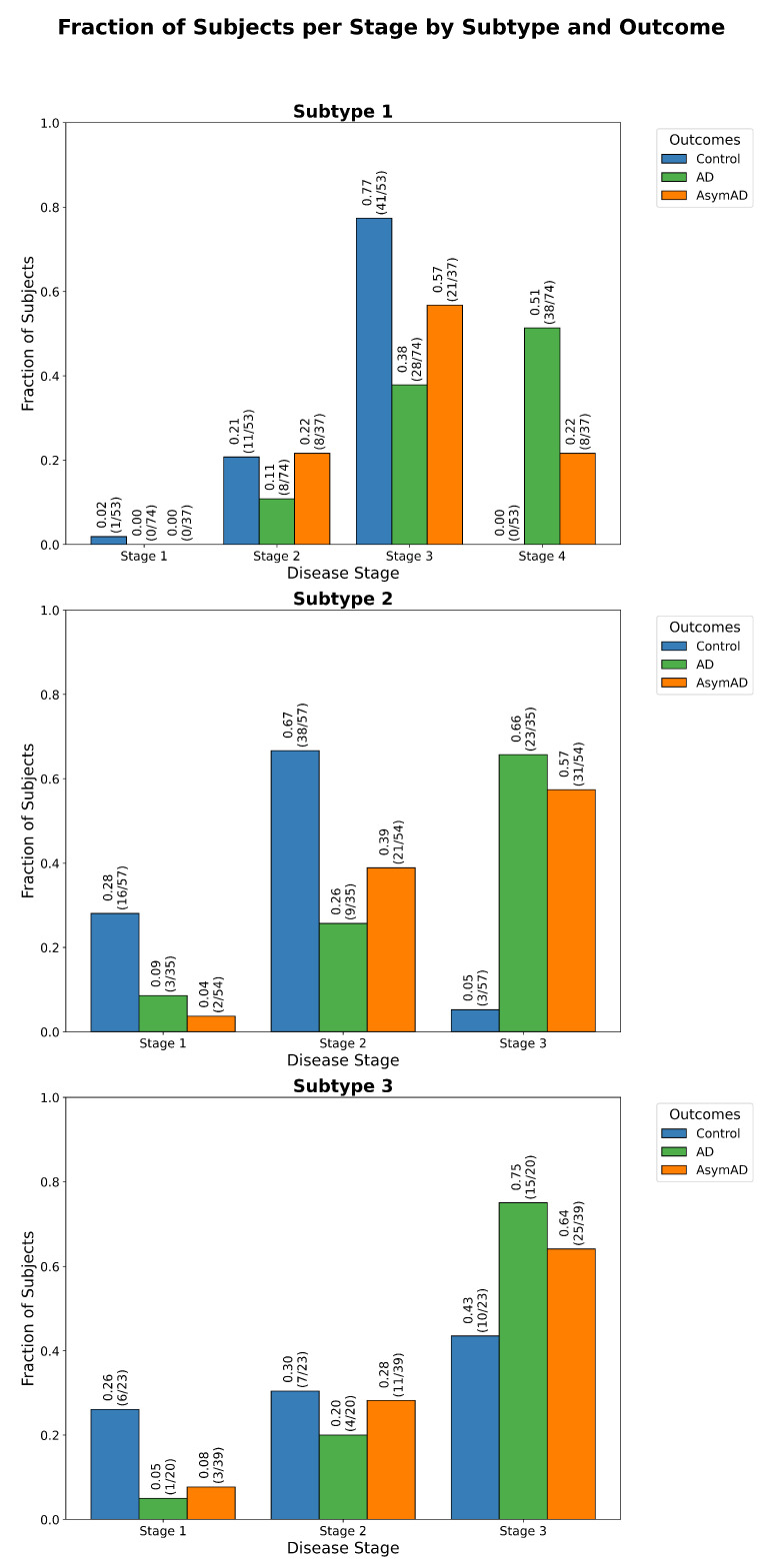
*Stage assignment across cognitive status groups.* Distribution of ReduXis-inferred disease stages across subjects from the EHBS study, stratified by cognitive status. Stage assignments are based on cerebrospinal fluid (CSF) peptide profiles using the same panel of 71 peptides from Figure 7. The observed distribution follows a biologically consistent progression: Control individuals are primarily assigned to early stages, AsymAD subjects exhibit a broader distribution spanning intermediate stages, and AD individuals cluster in late stages. A $\chi^{2}$ goodness-of-fit test (p<0.01), detailed in Table A6, confirms that the stage distributions are highly unlikely to have occurred under a null hypothesis of random assignment. This implies that ReduXis’s staging reflects meaningful biological differentiation across cognitive subtypes.

**Figure 9:**
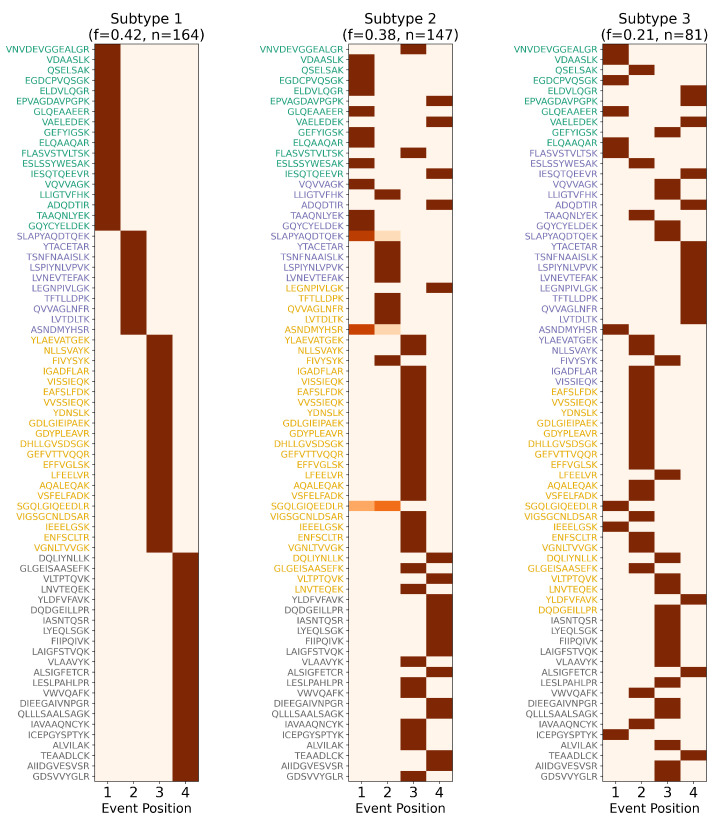
*Subtype-specific staging of Alzheimer’s disease biomarkers*. Event-based progression matrices showing ReduXis-inferred staging confidence for 71 cerebrospinal fluid (CSF) peptides across 3 distinct AD subtypes: Non-Inflammatory, Inflammatory, and Cortical Atrophy. As with previous staging matrices (see Figure 3 and Figure 6), color intensity represents confidence in stage assignment: deep maroon red indicates high confidence that a particular peptide exhibits altered expression early (Stage 1) or late (Stage 4) in the subtype’s inferred trajectory, while lighter shades indicate greater uncertainty. Despite the inherent complexity of subtype-specific molecular dynamics, ReduXis assigns stage transitions with high confidence across peptides, enabled by robust data preprocessing using [Q3 + 1.5 × IQR] clipping and rescaling to neutralize the influence of outliers. This standardization ensures that stage inference reflects the collective signal across all 71 peptides, not just a few dominant features. These patterns are congruent with known biological divergence across AD subtypes, which differ in anatomical burden, onset patterns, and progression rates. Collectively, this matrix underscores the strength of ReduXis in uncovering temporally structured, subtype-specific biomarker dynamics from CSF proteomics data, providing insights into the molecular heterogeneity underlying AD.

These findings highlight ReduXis’s capacity to deliver temporally nuanced, subtype-aware staging from CSF proteomic data. From capturing global biomarker gradients (Figure 7), to mapping individualized disease trajectories (Figure 8), to resolving subtype-specific progression patterns (Figure 9), the method provides a powerful framework for understanding molecular heterogeneity in AD. Furthermore, statistical validation of stage separability, stratified by subtype and clinical outcome, is shown in Table A6. $\chi^{2}$ analysis revealed statistically significant differences (p<0.01) in most subtype-stage combinations, confirming the robustness of ReduXis’s disease modeling. Finally, the molecular subtypes derived from EHBS CSF proteomic data largely mirror the progression trends previously identified using imaging-based subtyping via s-SuStaIn on the ADNI cohort [9], further validating the cross-modality consistency of these disease trajectories.

### 2.4. Interpretation of Consistency and Reproducibility

Across all evaluated datasets, the ReduXis EBM framework demonstrated consistent event sequencing and reproducible biomarker prioritization, underscoring its robustness in heterogeneous clinical and molecular settings. The ensemble-based voting strategy for feature selection resulted in low variance among selected features, with an average pairwise feature overlap exceeding 70%. This high degree of overlap indicates strong convergence across independent cross-validation folds.

Subtype and stage assignments remained stable across repeated bootstrapped runs, further supporting the reliability of the model’s classifications. Importantly, the inferred ordering of molecular events exhibited high consistency even under aggressive feature reduction protocols, highlighting the method’s resilience to input dimensionality constraints.

As shown in Table 1, the average feature stability score exceeded 0.615 across all datasets. This metric—calculated from the frequency with which a feature was selected among the top *K* features across three classifiers and five cross-validation folds (i.e., 15 total iterations)—indicates that, on average, selected features consistently appeared in at least 9 out of 15 runs. Remarkably, even for the most challenging dataset, derived from the TCGA-BLCA project—a 3-class classification task lacking a clearly defined control group—the feature selection stability score remained above this conservative threshold, underscoring the robustness and reproducibility of the selected biomarkers across diverse experimental conditions.

In contrast, higher stability was observed in less complex or more structured datasets. The colorectal cancer dataset (binary classification) achieved an average stability score of 0.615, while the EHBS dataset—which did not require feature selection due to its constrained dimensionality—achieved perfect consistency (stability score = 1.000). Although excluded from feature selection benchmarking, this result further illustrates ReduXis’s robustness in scenarios with minimal variability.

Compared with standard pipelines using s-SuStaIn alone [8], ReduXis offered multiple practical advantages, particularly evident in the multifaceted EHBS dataset:

1. **Runtime Efficiency via Feature Capping:** ReduXis introduces an ensemble-based feature selection strategy that constrains the final input to a tractable number of high-value biomarkers. By limiting the number of features prior to model fitting, ReduXis shifts computational complexity from being feature-dominated to being primarily dependent on the number of iterations and subtypes—substantially improving usability and scalability.
2. **Enhanced Intermediate Transparency:** The inclusion of intermediate outputs—such as confusion matrices, classification reports derived from LightGBM-based classification, and schema validation checks—enhances interpretability and facilitates the tracing of model behavior, while still requiring domain-specific expertise for accurate contextualization.
3. **Human-Readable Output:** The presentation of results in accessible tabular formats (e.g., CSV, PNG)—encompassing predicted stage assignments across subtypes and biomarker-specific disease trajectories—improves usability for downstream analysis and enables more effective validation. These tabular outputs are generated via the export of dataframes containing per-subject and per-biomarker inferences regarding stage and subtype, thereby efficiently conveying the interrelationship among assigned stage, assigned subtype, disease outcomes, and disease trajectories.

While minor variability was observed in deeply imbalanced classes (e.g., the “Normal” group in colorectal adenocarcinoma or the “Localized” group in transitional cell carcinoma), such variability is expected in small-sample, high-dimensional spaces and reflects real-world data imbalance. ReduXis mitigated these effects using a combination of sample weighting and threshold-based pruning strategies. Specifically, the pruning threshold was defined using a stability score, calculated as the proportion of times a given feature appeared among the top K most important features across 5-fold cross-validation repeated over three classifiers (yielding 15 model evaluations). Features with stability scores below a threshold were excluded. The threshold range of 0 to 1 was empirically selected, consistent with commonly used benchmarks for feature selection stability—where 0 indicates no reproducibility across folds, and 1 reflects ideal consistency across folds. This stability metric parallels the rationale of rank-based measures such as Kendall’s tau, where reduced variation across folds corresponds to increased reliability. Collectively, these results demonstrate that ReduXis maintains strong reproducibility and temporal consistency even in the presence of substantial biological and cohort-level heterogeneity, supporting its use in biomarker discovery and progression modeling across diverse clinical scenarios.

### 2.5. User Guidance and Built-In Safeguards

**Figure A1:**
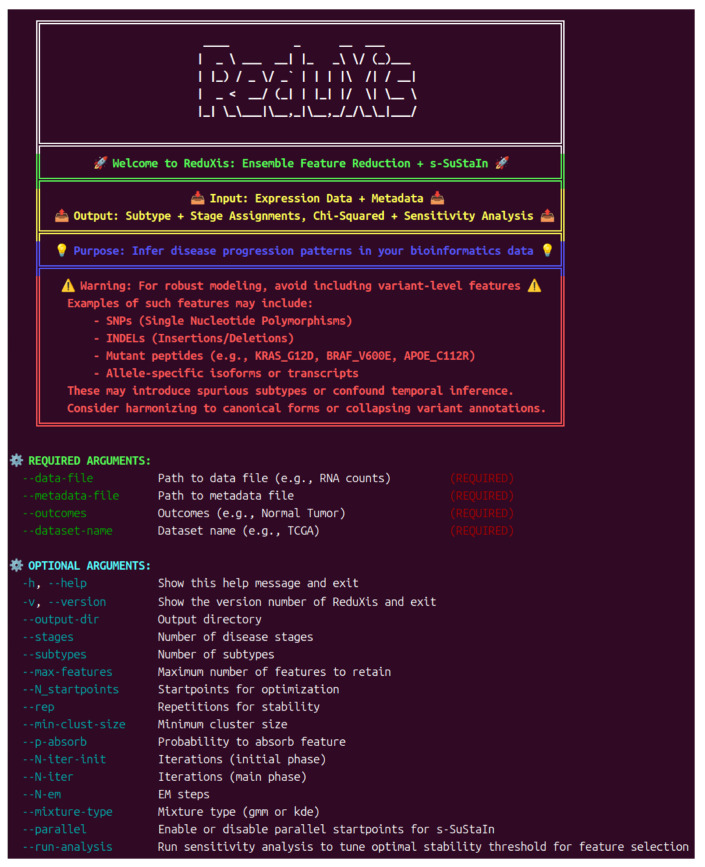
*ReduXis welcome screen and dashboard overview.* The ReduXis interface provides a user-friendly, visually intuitive dashboard upon launch. Key functionalities—such as dataset import/export, feature selection, and classification workflows—are clearly organized. The design emphasizes accessibility, employing informative tooltips and clear visual cues to guide users through the analysis pipeline, particularly beneficial for first-time users managing complex biomedical data.

ReduXis is designed to be accessible to non-expert users. Not only does it incorporate multiple failsafe mechanisms that promote responsible usage and reduce the likelihood of spurious or uninterpretable results, but it also has a user-friendly, interactive command-line interface (CLI), as shown in Figure A1, that enhances usability through intuitive design. It employs clear, color-coded prompts and messages—such as green for informational updates, yellow for warnings, and red for errors—following a traffic light-inspired scheme to facilitate quick interpretation. The CLI also incorporates built-in guidance: when required arguments are missing, the system explicitly prompts the user to provide them, rather than immediately terminating with an error. This structured interactivity reduces user error and promotes efficient, accurate use of the tool.

However, the effective application of this framework still benefits from domain expertise, particularly when choosing the number of stages and subtypes to ensure biologically coherent and interpretable outcomes. To balance flexibility with robustness, ReduXis introduces both “hard” constraints and “soft” defaults that guide user input while aligning with established scientific precedents. More specifically, the framework enforces a maximum of 150 features, five subtypes and six stages, reflecting common boundaries in molecular subtyping literature. Additionally, it sets a soft lower limit of 10 biomarkers per cluster, encouraging stability and biological plausibility in the resulting modules. These design choices are rooted in both empirical studies and widely accepted domain knowledge, ensuring that the model remains both practically usable and biologically grounded.

#### 2.5.1. Maximum Number of Biomarkers

ReduXis imposes an empirically derived upper limit of 150 biomarkers to mitigate excessive runtime in its event-based disease progression model. This constraint arises from the model’s reliance on inferring an optimal ordering over *M* biomarkers—a process that scales linearly with respect to *M*. When modeling *M* biomarkers across *N* subjects, *K* subtypes, *S* stages, and over *I* EM iterations, the algorithm’s total runtime can be expressed as:

$$O\left(\left(I_{EM}+I_{MCMC}\right)\times N\times K\times S\times M\right)+O\left(I_{EM}\times K\times M^{2}\right)$$

This complexity reflects the standard expectation-maximization (EM) algorithm’s structure:

- **E-step:** Computes assignment probabilities for all subjects, stages, and subtypes over biomarker orderings, contributing a runtime of $O(I_{EM}\times N\times K\times S\times M)$.
- **M-step:** Involves sorting biomarkers, thereby updating disease trajectories across subtypes, contributing $O(I_{EM}\times K\times M^{2})$.
- **MCMC step:** Involves a "burn-in" step and a "final iteration" step, reevaluating likelihoods across all subjects, stages, and subtypes, contributing $O(I_{MCMC}\times N\times K\times S\times M)$

Under the assumptions that *I* is constrained (i.e., convergence occurs quickly), *N* is fixed (i.e., number of subjects in the experiment or dataset of interest), K≤5 subtypes and S≤6 stages as software-enforced hard limits, the E-step dominates the runtime, and the overall complexity becomes effectively quadratic in *M*:

$$\mathrm{Runtime}\approx O(M^{2})$$

Given the steep scaling of runtime with increasing biomarker count—especially due to the internal ordering process—ReduXis enforces a hard cap of 150 biomarkers to ensure computational feasibility in practice.

#### 2.5.2. Maximum Number of Subtypes

Well-established subtyping frameworks in oncology and complex diseases rarely exceed five groups. For instance, in breast cancer, intrinsic molecular subtypes such as luminal A, luminal B, *HER2*-enriched, basal-like, and occasionally normal-like or claudin-low have consistently been validated across diverse cohorts [20,21]. As such, limiting the maximum number of subtypes to 5 provides a biologically grounded cap that captures established heterogeneity while preventing over-fragmentation, especially in moderate-sized datasets where overfitting can be a concern.

**Figure A2:**
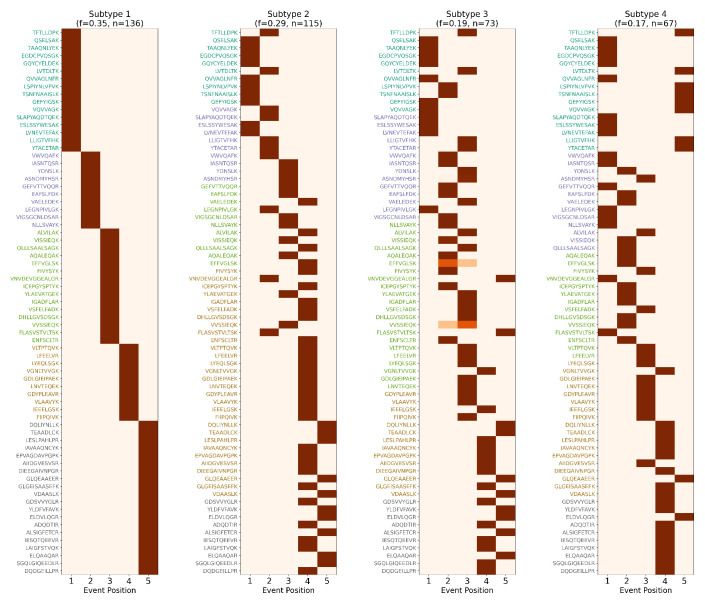
*Granularity of event-based models upon excessive inferred stages and subtypes.* This visualization uses the same EHBS dataset as Figure 9, but differs critically in the user-specified parameters: here, s-SuStaIn was configured with 5 stages and 4 subtypes, as opposed to the more conservative 4 stages and 3 subtypes. This small increase in input flexibility leads to a disproportionately large impact on model interpretability and robustness. By forcing s-SuStaIn to distribute progression trajectories across more stages, the model is pressured to “stretch” its inference over finer-grained temporal resolution, leading to smaller and less coherent biomarker clusters. This makes individual stage transitions more susceptible to stochastic variation or noise, especially in subtypes with fewer represented subjects. In addition, with an extra inferred subtype, the algorithm searches for subtle trajectory differences that may not be biologically meaningful, increasing the risk of overfragmentation and false discovery. The resultant noisy patterns often reflect sparse subject assignment or forced separation of weakly supported signals, undermining biological interpretability. This stark contrast underscores the importance of domain expertise in selecting modeling parameters. Without careful input, automated modeling can easily generate visually complex but biologically ambiguous outputs, highlighting the need for informed constraint-setting in unsupervised discovery frameworks like ReduXis.

Interpretation, however, still benefits from domain expertise. For example, Figure A2 shows the same EHBS dataset as in Figure 9, but with five stages and four subtypes passed instead of four stages and three subtypes. This leads to increased granularity, as the event-based model must stretch its inference across 71 biomarkers into more stages. Usually, when the event-based model is run, it sorts the given biomarkers by inferred stage. However, when the number of given subtypes is too high, this “staircase” visualization is only observed in the first couple of subtypes, and is lost completely in later subtypes where subject representation becomes sparse, highlighting the trade-off between resolution and interpretability.

#### 2.5.3. Minimum Cluster Size

A minimum cluster size of 5, with a default threshold of 10, was chosen to strike a balance between biological plausibility and statistical reliability. Whereas users are welcome to tune the minimum cluster size to something other than 10, it is highly recommended not to tune the cluster size so high as to cause issues with stage sizing, and it is required to keep the cluster size above 5 to ensure adequate modeling complexity. Many molecular pathways involved in disease progression consist of interacting modules of roughly 5–10 genes or proteins. For example:

1. The PI3K/AKT/mTOR pathway in cancer involves six core components [22].
2. Wnt signaling, central in development and tumorigenesis, also features clusters of 5–10 interacting biomolecules [23].
3. In neurodegenerative diseases, protein-protein interaction subnetworks of 5–8 key molecules often define functional modules [24].

From a modeling standpoint, setting a lower bound of 5 prevents spurious or noisy clusters from forming, while the default of 10 ensures that the resulting subtypes or clusters are biologically meaningful and statistically robust—particularly critical in high-dimensional omics settings where false discoveries are a known risk.

#### 2.5.4. Maximum Number of Stages

Classical staging systems also inform our choice of stage granularity. The AJCC TNM framework stratifies many solid tumors into five clinical stages (0–IV), with occasional A/B subdivisions but rarely exceeding six total categories [25]. In neurodegeneration, Braak’s neuropathological schema describes six stages of tau deposition in AD [26]. Although imaging-based event models may collapse these into fewer clinical stages, capping at 6 ensures compatibility with canonical frameworks.

#### 2.5.5. Safeguards Against Overparameterization

Users must explicitly define the number of subtypes and stages prior to modeling. This is necessary because unsupervised estimation of these parameters is underconstrained in high-dimensional clinical datasets, often resulting in overfitting or biologically implausible solutions. Prior work has emphasized that overparameterization in subtype models can lead to fragmentation and instability, especially when sample sizes are modest [2,6]). For this reason, ReduXis includes multiple safeguards:

1. **Subtype Sanity Checks:** If users define more subtypes than the dataset can reasonably support, which is defined as having subtypes with fewer than 10 subjects, the pipeline automatically merges sparsely populated subtypes into the last valid one. A warning message is issued to ensure transparency.
2. **Stage Density Filtering:** When an excessive number of stages is specified, such as 10 stages, ReduXis filters out sparse stages. Stages containing fewer than three subjects and only a single outcome class are eliminated and excluded from final visualizations to prevent over-interpretation.
3. **Format Validation:** As outlined in the Section 2 strict data format and column naming conventions are enforced to prevent silent errors. The system halts execution if violations are detected, providing actionable error messages.
4. **Visual Diagnostics:** The output includes staging bar plots and stratified heatmaps per subtype. These visualizations help users detect potential modeling failures, such as flat staging distribution, automatic assignment to the final stage due to a lack of monotonic change, or no class separation, promoting manual review before interpretation.

By anchoring subtype and stage limits in established frameworks and embedding automated checks, ReduXis promotes robust, interpretable disease models. Users should review post hoc assignment distributions—non-uniform, progressively ordered changes across stages and clear subtype differentiation indicate a well-specified model, whereas homogeneous patterns may warrant re-evaluation of feature selection or parameter choices [2,6].

## 3. Materials and Methods

This section details the case study data sources and the ReduXis design criteria, development, implementation, and evaluation metrics.

### 3.1. Overview of ReduXis Framework

ReduXis builds upon the open-source pySuStaIn [6,7] and s-SuStaIn [8] frameworks to implement event-based models (EBMs) for simultaneous subtype and stage inference. EBMs infer a probabilistic temporal ordering of biomarker transitions—conceptualized as sequential “events” from a normal to an abnormal state—using cross-sectional data. Each subject is probabilistically assigned to a disease stage and subtype based on likelihood estimates of having undergone each biomarker event in the inferred sequence.

**Figure 10:**
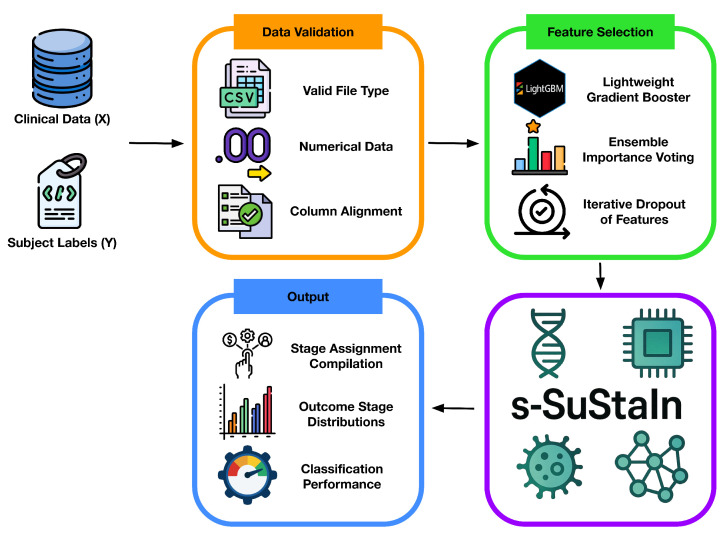
*Concept sketch of the ReduXis pipeline.* ReduXis extends s-SuStaIn [8] by incorporating (i) robust data-integrity checks during preprocessing to detect and handle missingness or label imbalances, (ii) ensemble-based feature selection when dimensionality exceeds a user-defined threshold, and (iii) an export layer that automatically generates transparent, interpretable outputs—including tabular subtype/stage assignments, annotated progression plots, and intermediate diagnostics—to improve accessibility and reproducibility. In contrast to pySuStaIn and s-SuStaIn, which offer limited output interfaces primarily through internal object attributes or Python method calls, ReduXis prioritizes out-of-the-box interpretability by exporting human-readable outputs aligned with best practices in reproducible machine learning.

As shown in the schematic overview of the ReduXis pipeline in Figure 10 below, our enhancements to pySuStaIn and s-SuStaIn emphasize user interactivity, automated summarization, and customizable visualization—features not present in the base implementation. Detailed design criteria, methods, parameters, and analysis are contained within this section below. Briefly, the principal modifications include the following:

1. **Real-Time Preprocessing Validation:** Automatic checks for data and metadata integrity (see Data Validation selection for more details), as well as immediate feedback on missing values, formatting errors, and schema violations.
2. **Seamless Integration with Ensemble-Derived Feature Space:** Automatic ingestion of a reduced feature set from the ensemble voting scheme (see Feature Selection section for more details), with a dynamic adjustment of EBM inputs based on classifier consensus.
3. **Customizable, User-Friendly Outputs:** ReduXis is designed to maximize transparency by exporting a comprehensive suite of intermediate and final outputs in standardized formats (e.g., CSV, PNG) to a user-specified directory. In contrast to pySuStaIn and s-SuStaIn—which require users to access internal Python object attributes or invoke specific methods to retrieve subtype assignments, biomarker trajectories, or model diagnostics—ReduXis automates this process and externalizes key layers of the modeling pipeline. This explicit output design eliminates the need for post hoc scripting and facilitates reproducibility, inspection, and secondary analyses. The exported outputs include the following:
  - **Subject- and Biomarker-Level Subtype/Stage Annotations:** CSV files detailing subtype and stage assignments at both the subject and biomarker levels, allowing easy integration with clinical cohorts or external validation data. In addition, these CSV files are in a machine-readable format suitable for downstream tasks such as survival modeling, classification, or longitudinal analysis.
  - **Subtype-wise Stage Distribution Plots:** Bar plots visualizing the distribution of disease stages within each subtype, stratified by clinical outcomes (e.g., “Localized,” “Intermediate,” “Metastatic”), enabling visual comparison of progression patterns across disease phenotypes.
  - **Biomarker Heatmaps by Stage and Outcome:** Heatmaps showing mean expression levels of selected biomarkers across disease stages and clinical subgroups, supporting rapid identification of stage-specific or outcome-linked molecular signatures.

### 3.2. Data Sources

This study incorporates three independent, high-dimensional omics datasets spanning neurodegeneration and oncology. The neurodegenerative cohort derives from the Emory Healthy Brain Study (EHBS), comprising cerebrospinal fluid (CSF) proteomic profiles with associated clinical metadata, previously shown to capture early AD biomarkers [18]. The oncologic datasets consist of bulk RNA sequencing (RNA-seq) matrices from The Cancer Genome Atlas (TCGA), accessed via the Genomic Data Commons (GDC) Data Portal. Specifically, we analyzed (i) a case–control cohort of colorectal adenocarcinoma patients and (ii) a cohort of transitional cell carcinoma subjects stratified into distinct clinical stages to represent progressive disease states. Collectively, these datasets exemplify the heterogeneous, high-dimensional biomolecular landscapes characteristic of chronic, progressive diseases.

### 3.3. Design Criteria

The development of ReduXis was guided by a set of pragmatic and principled design criteria aimed at overcoming key limitations in existing event-based modeling frameworks, particularly the pySuStaIn [7] and s-SuStaIn [8] implementations. Our objective was to construct a robust, generalizable, and user-friendly platform capable of handling high-dimensional biomedical datasets with minimal manual intervention, while preserving the interpretability and rigor of the underlying probabilistic models. The key design principles are outlined below:

1. **Rigorous Data Validation:** Biomedical datasets are often noisy, incomplete, or inconsistently formatted. To address this, ReduXis incorporates a robust preprocessing pipeline that enforces schema conformity, flags missing or malformed entries, and ensures the integrity of both input matrices (predictors *X* and labels *Y*). This validation step guarantees that downstream modeling is performed on clean, well-structured data.
2. **Standardized Preprocessing for EBM Compatibility:** The EBM framework requires strictly numerical and tabular input with bounded, non-negative values and no extreme outliers. To this end, ReduXis automates common preprocessing tasks—normalization, outlier filtering, type coercion, and data harmonization—thereby reducing user burden and increasing consistency across datasets.
3. **Flexibility and Scalability to High-Dimensional Data:** Traditional EBM implementations are optimized for low-dimensional neuroimaging cohorts (e.g., ADNI), limiting their utility in broader biomedical contexts such as oncology or multi-omics studies. ReduXis was, therefore, designed to ingest complex, high-dimensional tabular data, with built-in dimensionality reduction via ensemble-based feature selection to maintain tractable model performance.
4. **Transparent and Interpretable Outputs:** A core limitation of existing EBM tools is their “black-box” nature, which hampers clinical interpretability. ReduXis addresses this by exporting both tabular and graphical outputs—including biomarker heatmaps, stage-wise subtype distributions, and subject-level annotations—that allow users to visually and quantitatively inspect model behavior. This enables distinction between biologically meaningful stage transitions and model artifacts, such as biomarkers erroneously clustered at terminal stages due to a lack of dynamic range.
5. **User-Centric Interface and Automation:** To facilitate adoption by clinical researchers and data scientists, ReduXis features an interactive CLI with informative prompts, real-time validation feedback, and customizable output settings. This enhances user engagement and reduces the barrier to entry for non-technical users.
6. **Open-Source and Python-Native Implementation:** By building on a widely adopted language in the biomedical and data science communities, ReduXis ensures accessibility, extensibility, and ease of integration into existing workflows. Its modular design allows researchers to adapt the pipeline to specific research questions while benefiting from community-driven development.

Collectively, these design criteria reflect a commitment to creating a practical, extensible, and interpretable modeling framework tailored for modern biomedical datasets, while preserving the statistical rigor of event-based disease progression modeling.

### 3.4. Data Validation and Preprocessing

**Figure 11:**
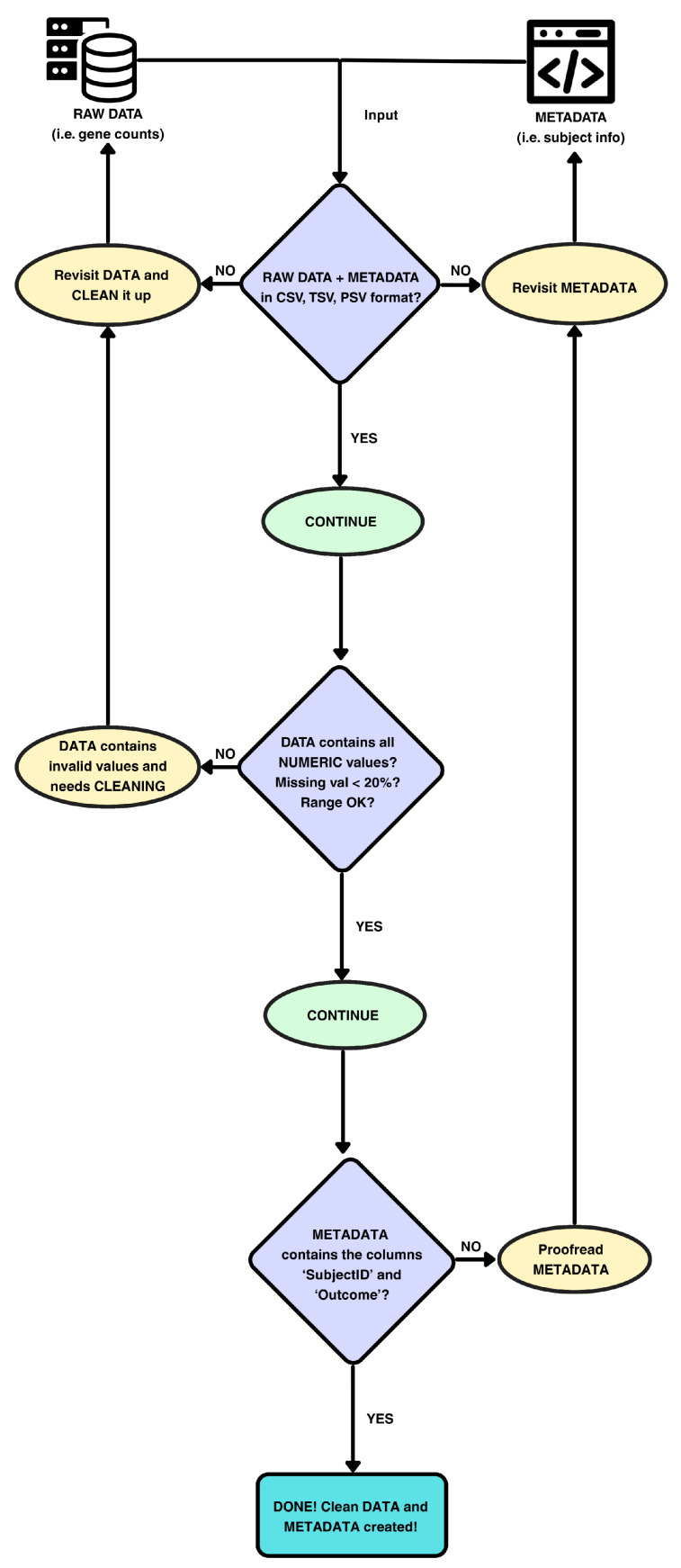
*Schema of data preprocessing and internal checks in ReduXis.* ReduXis requires 2 inputs: (1) the main data matrix (biomarker measurements) and (2) accompanying metadata. Unlike previous methods, ReduXis implements automated data-validation checks prior to s-SuStaIn, including schema enforcement, missingness filtering, and outlier capping, thereby minimizing user-induced errors and ensuring robust downstream modeling.

Prior to EBM fitting, ReduXis performs automated validation and preprocessing to ensure data readiness, as shown in Figure 11 below. These steps safeguard downstream analyses, enhance reproducibility across reruns and datasets, and reduce the preprocessing burden on end users. All violations of data integrity—formatting errors, schema mismatches, or outlier conditions—are flagged through real-time user feedback.

The following steps are conducted in order to ensure that the data that is fed into the EBM is valid and cleaned up:

1. **File Format Checks:** Both data and metadata must be in a tabular format (CSV, TSV, or PSV).
2. **Numeric Enforcement:** All entries in the data matrix are required to be numeric (integer or floating-point).
3. **Schema Enforcement:** Metadata must contain at least two columns:
  - **SubjectID:** Unique identifier for each subject, used to align metadata with the data matrix via exact matching.
  - **Outcome:** Clinical outcome label (e.g., case/control or disease stage) required for supervised analyses.
4. **Missingness Filtering:** Features with >20% missing values are excluded, while those with ≤ 20% missing values are imputed using k-nearest neighbors imputation (kNN, k = 5).
5. **Outlier Capping:** Values > Q3 + 1.5 × IQR are capped at Q3 + 1.5 × IQR, where Q3 and IQR denote a particular feature’s third quartile and interquartile range, respectively.

### 3.5. Feature Selection via Ensemble Dimensionality Reduction

**Figure 12:**
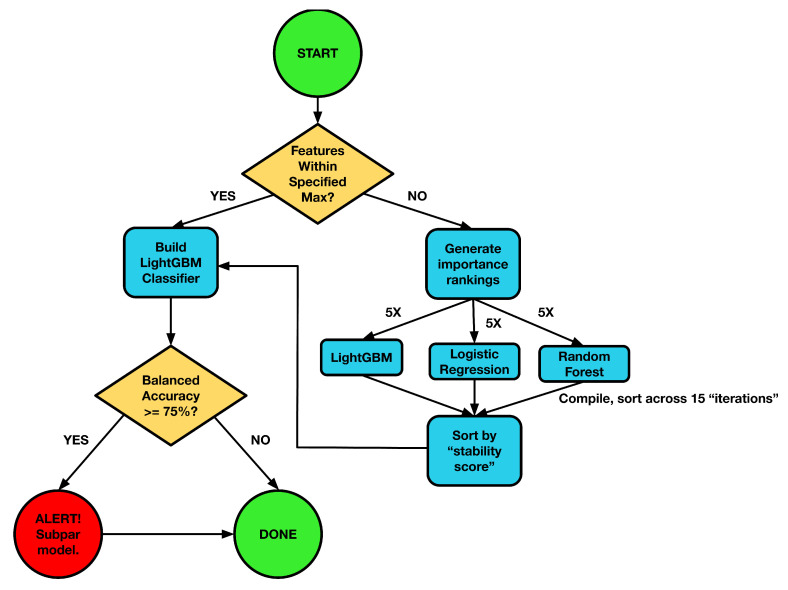
*Schema of ReduXis feature selection.* Three classifiers, light gradient boosting (LightGBM), logistic regression, and random forests [11,12,27], are independently trained on the full dataset. Class imbalance is corrected via sample weighting and imbalanced learning through SMOTE oversampling. After training, each model’s feature-importance scores are extracted. The top-*K* features from each model form candidate sets; features that score the highest “stability score” are ranked highest and are preserved for model fitting. This stability-based filtering ensures stability and robustness against model-specific noise.

High-dimensional omics datasets (often with 1000–10,000 features) pose a significant risk of overfitting and unstable feature selection. To mitigate this, ReduXis employs a supervised ensemble feature selection approach for dimensionality reduction prior to EBM fitting, using a nested cross-validation (CV) framework to prevent data leakage or circularity, as shown in Figure 12 below. Specifically, feature selection was performed independently within each of five cross-validation folds, using an ensemble of three classifiers—LightGBM, logistic regression, and random forest—to rank features. To ensure reproducibility and independence, we initialized the ReduXis pipeline with a fixed base random seed of 42, incremented by 1 for each fold (e.g., fold 1 = 42, fold 2 = 43, etc.). The top *K* features from each classifier and fold were aggregated, and a final feature stability score was computed to quantify the frequency with which features appeared across runs. The most consistently selected features were retained for EBM training. Importantly, EBM model performance and tuning were never used in the feature selection process, ensuring a clear separation between feature ranking and final model evaluation.

**Figure A3:**
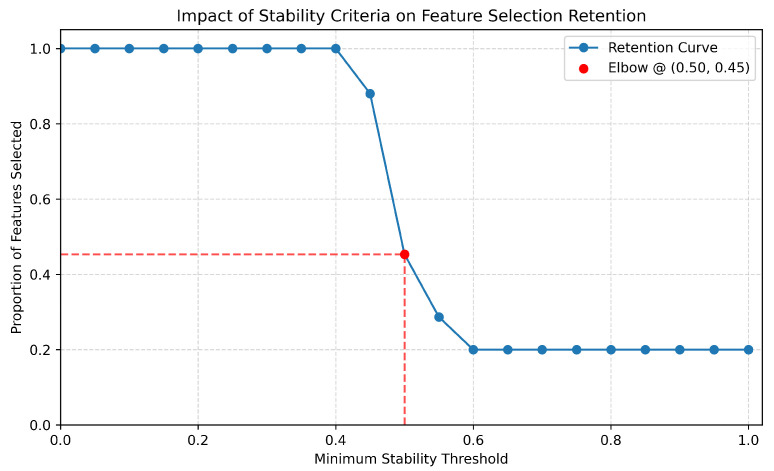
*Determination of the optimal stability threshold for feature retention.* This plot demonstrates how the number of retained features varies as a function of the minimum stability threshold. Rather than evaluating model performance (e.g., sensitivity or specificity), the focus here is on identifying a threshold that balances feature robustness with inclusion rate. Using an elbow-point heuristic, we locate a threshold—specifically, 0.5—where the rate of change in retained features (Δy) significantly shifts. Thresholds below this point retain too many unstable features (overly permissive), while thresholds above become overly restrictive, excluding potentially informative variables. The selected threshold thus represents a principled compromise for robust downstream analysis.

**Figure A4:**
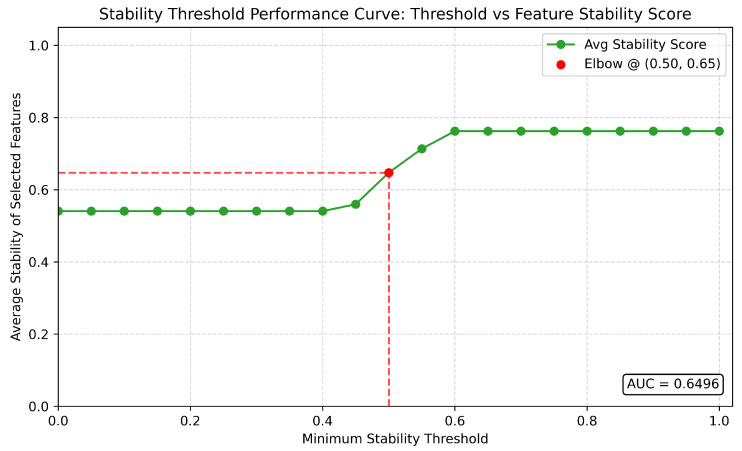
*Quasi-sensitivity–specificity analysis for stability threshold validation.* While not a formal ROC curve, this figure presents a quasi-analytical framework mimicking sensitivity–specificity dynamics to validate the selection of a minimum stability threshold. The x-axis reflects varying stability thresholds (0–1), and the y-axis represents derived metrics including average and minimum feature stability—serving as analogues to sensitivity and specificity in this context. Consistent with Figure A3, an inflection point or “elbow” is again observed at a threshold of 0.5, reinforcing its selection as an optimal trade-off between retaining too many unstable features and applying overly strict filtering. Notably, the average stability score also peaks in curvature at this threshold, adding further confidence. Additionally, the minimum stability remains above 0.5 across all thresholds (AUC = 0.6495), suggesting that the dataset is inherently robust and relatively insensitive to extreme thresholding. This dual-figure approach provides both exploratory and confirmatory support for the chosen parameter.

Moreover, the stability of feature selection is quantified using a custom, easy-to-understand “scoring system” that works like this:

1. **Determination of *K*:** Let *p* = total number of features in the dataset. *K* is set to 5% of *p*, or 3 times $U_{\max}$, where $U_{\max}$ is the user-specified maximum number of final features. We choose three times the user-specified maximum (rather than the maximum itself) to account for model-specific noise in the top-*K* ranking. Finally, *K* cannot exceed *p* itself.
2. **Classifier Training and Importance Extraction:** Each classifier—Light Gradient Boosting Model (LightGBM), Logistic Regression, and Random Forest—is trained using 5-fold cross-validation with sample weights to correct for class imbalance. Feature-importance scores are extracted from each model (e.g., gain for LightGBM, coefficient magnitude for Logistic Regression, and mean decrease in impurity for Random Forest).
3. **Ensemble Importance–Based Filtering:** For each classifier and fold, the top-*K* features (ranked by importance) are recorded. A feature is selected if it meets one of the following criteria:
  - It has a stability score >0.5 (high stability), or
  - It appears among the top-*N* most stable features, where *N* is the user-chosen final feature count.
  To elaborate on the three possible scenarios:
  - If more than *N* (but up to *K*) features have stability scores >0.5, then the model chooses the top *N* by stability ranking.
  - If fewer than *N* features have stability scores >0.5, but at least one feature does, the model retains all features with stability scores >0.5 and then “clips” additional features—ranked by descending stability—until either the lowest stability score of the retained set exceeds 0.5 or the total feature count reaches the minimum required for EBM training.
  - If all features have stability scores <0.5, then the model simply takes the top *M* features with the highest stability scores, where *M* is the minimum number of features needed to meaningfully cluster and run the EBM given the user-defined constraints (e.g., minimum features per stage and number of stages).
  This ensemble voting mechanism ensures that no single classifier’s idiosyncrasies dominate feature selection.
4. **Threshold Rationale:** The stability score for each feature *f* is defined as
  $$S\left(f\right)=\frac{\mathrm{Number}\;\mathrm{of}\;\mathrm{times}\;f\;\mathrm{appears}\;\mathrm{in}\;\mathrm{the}\;\mathrm{top}-K\;\mathrm{lists}}{\mathrm{Total}\;\mathrm{possible}\;\mathrm{number}\;\mathrm{of}\;\mathrm{appearances}\;\mathrm{of}\;f\;\mathrm{in}\;\mathrm{top}-K\;\mathrm{lists}}\;\to \;S\left(f\right)\in \left[0,1\right].$$
  In our 5-fold cross-validation with three classifiers, each feature can appear at most 15 times among the top *K* lists. A threshold of 0.5, therefore, implies that a feature appears roughly 7 to 8 times out of 15, indicating a moderate degree of consensus across folds and classifiers. This specific cutoff was chosen after iterative tuning—evaluating range values from 0 to 1—so as to balance two competing goals:
  - Preventing overly aggressive pruning (which can discard genuinely informative features if the threshold is set too high), and
  - Avoiding the unintentional inclusion of features with low reproducibility (which can occur if the threshold is too low).
  Through this analysis, as shown in Figure A3 and Figure A4, we found that the threshold of 0.5 achieves a practical trade-off between sensitivity (adequately stable features can still be considered) and specificity (only highly reproducible features pass). In addition, Figure A4’s depiction of not only having an “elbow” point at 0.5, just like with Figure A3, but also having an AUC of approximately 0.65 indicates both robustness of feature stability and reinforces the suitability of the 0.5 threshold as an inflection point, beyond which additional gains in feature stability are marginal. This suggests that 0.5 serves as an optimal cutoff, balancing inclusivity of stable features with stringent reproducibility criteria.
5. **Stability Scoring:** For each feature *f*, the stability score S(f) is computed as above. Table 2 reports the average stability score for each of the three datasets used in this study.

After ensemble stability-based voting and filtering, the feature space in bulk RNA-seq datasets is reduced by 99%, greatly improving computational efficiency and convergence during EBM fitting.

### 3.6. Evaluation Metrics

In terms of classification performance, ReduXis evaluates stage- and subtype-prediction accuracy using standard metrics: precision, recall, and F1-score, all computed on the feature-reduced datasets. These results are automatically compiled into a comprehensive classification report. In addition to these conventional metrics, ReduXis also computes an ordinal accuracy score to better reflect performance on classification tasks where class labels are inherently ordered (e.g., disease stages). Unlike standard accuracy, which counts only exact matches as correct, ordinal accuracy penalizes misclassifications based on their distance from the true label.

This is achieved using a reward-based scheme defined by a distance-penalty matrix $R\in R^{K\times K}$, where *K* is the number of classes. The matrix entries are defined as follows:

$$R\left(i,j\right)=1-\frac{\left|i-j\right|}{K-1}$$

where *i* is the true class index and *j* is the predicted class index. Ideal matches yield a reward of 1, near misses receive a fractional reward, and the largest misclassifications yield a reward approaching 0. The final ordinal accuracy is computed as follows:

$$\mathrm{Ordinal}\;\mathrm{Accuracy}=\frac{1}{N}\sum_{i=1}^{K}\sum_{j=1}^{K}R\left(i,j\right)\cdot C\left(i,j\right)$$

where C(i,j) is the confusion matrix with raw counts of samples from the true class *i* predicted as class *j*, and *N* is the total number of samples. This formulation ensures that class imbalance is respected, and that model errors are penalized proportionally to their ordinal distance.

**Figure A5:**
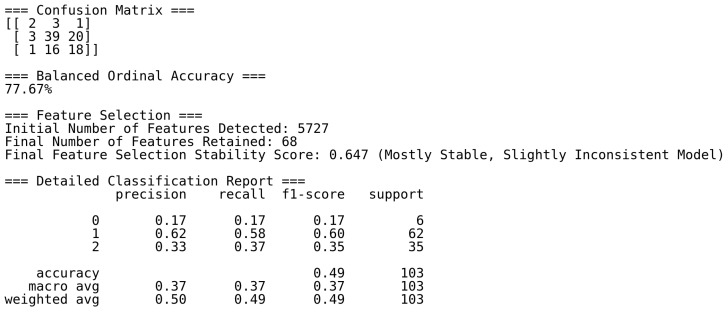
*Automated classification report for the TCGA transitional cell carcinoma dataset.* This report summarizes key performance metrics—precision, recall, F1-score, accuracy, support, and mean feature stability score—across all classes. Although the overall ordinal classification accuracy reaches 78%, performance is disproportionately driven by the majority classes. The “Localized” class (n = 19) is poorly detected, and confusion between the “Localized” and “Invasive” (n = 207) classes reflects both overlapping feature space and severe class imbalance. Notably, class weighting and resampling techniques were already applied, indicating that this result represents the upper bound of achievable performance under current data constraints. These challenges underscore the difficulty of modeling minority classes in high-dimensional, low-sample-size contexts. Nonetheless, the robustness of the ReduXis pipeline—including ensemble-based feature selection and gradient-boosted decision trees—enables consistent performance across multiple datasets, even under such stringent conditions.

**Figure A6:**
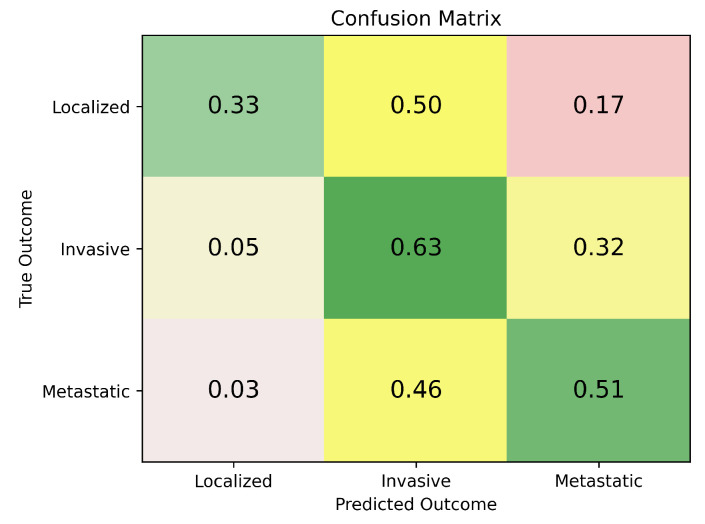
*Normalized confusion matrix for the TCGA transitional cell carcinoma dataset.* This figure visualizes ReduXis’ prediction accuracy across TCC histological grades (Localized: n = 19, Invasive: n = 207, Metastatic: n = 117), with values normalized to the [0, 1] range. Diagonal cells represent correct classifications, while off-diagonal cells capture misclassifications. Darker green shades indicate high confidence and correct predictions, yellow shades signify near-misses—where the predicted class was close to the actual label—and red shades highlight more substantial classification errors, where predictions diverged significantly from the ground truth. Unlike traditional accuracy metrics, balanced ordinal accuracy accounts for class imbalance by weighting performance by the frequency of each class, and assigns partial credit for predictions that are closer in ordinal space to the true class (e.g., misclassifying a high-grade tumor as medium-grade is penalized less than predicting it as low-grade). Together with Figure A4, which reports a balanced ordinal accuracy of greater than 75%, these results suggest that ReduXis maintains robust predictive power—even under challenging conditions. Notably, the model achieves this despite an aggressive dimensionality reduction from approximately 5700 initial features to just 68, and in the face of a pronounced 2:10:5 class imbalance across low-grade, medium-grade, and high-grade TCC samples. This highlights ReduXis’ strong generalization capabilities and sensitivity to subtle, grade-defining patterns in highly compressed multiomic space.

In addition, the “stability score”—defined as the average stability of a feature across all three classifiers and 5 folds (see the Feature Selection section)—is included in this report, as detailed in Figure A5. Furthermore, ReduXis generates a normalized confusion matrix that provides a detailed view of per-class classification performance (see Figure A6). This matrix is scaled from 0 to 1, where diagonal values represent correct classifications and off-diagonal values indicate misclassifications. The normalized values reflect the classification rate for each class, with an expected average accuracy of at least 75%. If the classification rate falls below this threshold, ReduXis issues a warning to indicate potential unreliability due to underperformance in classification.

To quantitatively assess the separability of stages across different subtypes and outcomes, ReduXis also computes a $\chi^{2}$ goodness-of-fit test. This test is stratified jointly by subtype and clinical outcome, with stages serving as the grouping variable, thereby forming a three-dimensional stratification. This design addresses potential class imbalance issues that can lead to inflated statistical significance when stratifying only by subtype. The null hypothesis assumes that stage assignment is random and uninformed by biomarker distributions. A significant $\chi^{2}$ result thus provides statistical evidence of stage distributional separation, quantifying the extent to which biomarker-driven stage assignments deviate from a random baseline and reflect meaningful biological or clinical stratification.

Feature selection consistency is assessed qualitatively through visual inspection of event ordering overlap across bootstrapped runs. In parallel, quantitative evaluation via the stability score measures the consistency of selected biomarkers across folds, classifiers, and random seeds. Despite the probabilistic nature of SuStaIn, ReduXis demonstrates strong repeatability of event sequences, with key disease-specific markers appearing consistently at similar stages across 10 independent runs with fixed hyperparameters.

To evaluate end-to-end efficiency, we compared ReduXis to a baseline workflow using standard s-SuStaIn with manual preprocessing. Wall clock time was recorded, encompassing data loading, preprocessing, feature selection, event-based modeling, and output generation, using internal logging tools integrated into ReduXis.

In these experiments, both workflows executed successfully. s-SuStaIn typically requires between 5 and 25 min of wall clock time, depending on data complexity and computational power, as shown in Table 3, Table 4 and Table 5. Furthermore, ReduXis has a similar runtime as s-SuStaIn. For the Emory Healthy Brain Study dataset (71 features), both ReduXis and s-SuStaIn required at least 1 h to complete. While the increased customizability of ReduXis may introduce modest variability in output sequences, this is generally outweighed by improvements in accessibility, reproducibility, and interpretability for most use cases. Despite the similar runtime between s-SuStaIn and ReduXis, ReduXis outperforms this prior software primarily due to more transparent preprocessing, detailed error handling, and well-documented requirements.

### 3.7. Software Implementation and Availability

ReduXis is distributed as a Python 3.12 package with a command-line interface (CLI), leveraging Colorama 0.4, Matplotlib 3.10, Numpy 2.2, Pandas 2.3, Scikit-learn 1.7, Seaborn 0.13, LightGBM 4.6.0, and an extended fork of pySuStaIn [6,7]. Python was selected for its widespread adoption in the biomedical and data science communities, facilitating installation, customization, and integration of the event-based modeling framework into existing analytical workflows. All computations were performed on a cloud instance with 384 GB of RAM and validated on standard desktop hardware (16 GB RAM) to ensure broad accessibility. A fixed random seed (seed = 42) was used for classifier training and event sequence generation to ensure reproducibility.

The ReduXis source code, documentation, and example workflows are available in an open-source, public GitHub (v1.0) repository (see Data Availability Statement). Additional materials, including preprocessing scripts and tutorial figures, are included in the same repository and further detailed in the Appendix A. The associated datasets are also described in the Data Availability Statement.

## 4. Conclusions

ReduXis addresses a critical unmet need in biomedical machine learning by delivering an end-to-end, interpretable, and reproducible framework for event-based modeling (EBM). While state-of-the-art tools like pySuStaIn and s-SuStaIn offer flexibility in subtype modeling, they often require expert-level knowledge for post hoc inspection and visualization. In contrast, ReduXis democratizes EBM through a fully integrated pipeline that includes (i) automated preprocessing checks, (ii) ensemble-based feature selection, and (iii) transparent, standardized outputs that are immediately accessible and coding-agnostic. These design choices lower the barrier to entry for computational biologists and translational researchers working with complex, high-dimensional data.

Across three domains—neurodegeneration, colorectal cancer, and bladder cancer—ReduXis demonstrated strong reproducibility, temporal consistency, and robustness to class imbalance and cohort-level heterogeneity. Classification efficacy and disease trajectory confidence held across datasets of varying size, signal-to-noise ratio, and feature count, suggesting generalizability to other clinical contexts. However, ReduXis is currently optimized for structured, quantitative omics data; its applicability to sparse, noisy, or categorical datasets remains an area for future validation.

### 4.1. Data Modality Compatibility

ReduXis is designed for quantitative, tabular datasets, particularly those derived from omics platforms such as RNA-seq, methylation arrays, and proteomics. It supports both unimodal and multimodal inputs structured in CSV, TSV, or PSV formats. It is not intended for unstructured or categorical modalities like clinical notes or radiology reports without preprocessing. Future iterations may include wrappers for converting common biomedical formats (e.g., DICOM, MAGE-TAB) into compatible structures.

### 4.2. Limitations and Future Directions

While ReduXis offers high reproducibility and interpretability, some limitations remain. The stability threshold for feature pruning is empirically chosen and may require dataset-specific tuning. Additionally, performance may vary with extremely small sample sizes or highly overlapping disease states, where EBM assumptions are challenged. Future work will address these limitations and expand capabilities in several directions:

1. **Domain Expansion:** Although validated on Alzheimer’s disease and two cancer types, ReduXis can be extended to other progressive disorders such as Huntington’s disease, ALS, or autoimmune conditions with longitudinal omics data.
2. **Clinical Integration:** With further validation, ReduXis could support real-time staging and subtype inference in clinical decision-support systems.
3. **Enhanced Multi-omics Support:** Future versions may include native integration for raw data formats and improved strategies for cross-modal feature harmonization.

In summary, ReduXis delivers a scalable, interpretable, and generalizable approach to temporal biomarker discovery. By bridging computational rigor with usability, it lays the groundwork for broader adoption of EBMs in both research and clinical pipelines. 

## Figures and Tables

**Table 1:** *Summary of datasets used.* Each dataset is derived from a distinct study or experiment, whose name is indicated by the **Dataset Name** column. The **Sample Size** column indicates the number of patients included. The **Biomarker Count** reflects the number of genes (in the case of TCGA datasets) or peptides (for EHBS). The **Composition** column describes the case-control makeup of each cohort, highlighting potential class imbalance.

Dataset Name	Sample Size	Biomarker Count	Composition
TCGA-COAD	446	5092	39 Normal, 407 Tumor
TCGA-BLCA	343	5727	19 Localized, 207 Invasive, 117 Metastatic
EHBS	392	71	133 Control, 130 AsymAD, 129 AD

**Table 2:** *Stability of feature selection across three datasets (5-fold CV, three classifiers) of top biomarkers.* Across all three case studies, one can see that there is a moderate degree of stability (score > 0.5), even with a small fraction of biomarkers selected from the TCGA datasets.

Dataset Name	Stability Score	Total Biomarkers	Biomarkers Selected
TCGA-COAD	0.615	5092	66 ^a^
TCGA-BLCA	0.647	5727	68 ^a^
EHBS	1.000 ^b^	71	71

^a^ Due to the quadratic increase in runtime associated with larger feature sets, the total number of features is automatically limited to 150 to maintain computational tractability. From this initial candidate set, features with stability < 0.5 are pruned, resulting in a diminished number of features. ^b^ No feature selection is performed because the CSF proteomics dataset contained only 71 features (<100); hence, stability is trivially 1.000.

**Table 3:** *Comparison of runtime across three datasets analyzed in this study.* Each dataset corresponds to a distinct external case study or experiment, identified in the **Dataset Name** column. The **Stages**, **Subtypes**, and **Features** columns indicate the number of disease stages and subtypes provided as inputs to ReduXis, as well as the number of input features (e.g., biomarkers or imaging variables) in each dataset. The **s-SuStaIn Wall Time** and **ReduXis Wall Time** columns report the wall times for the legacy s-SuStaIn method and the ReduXis framework, respectively. While s-SuStaIn scales poorly with the number of input features, ReduXis achieves substantial computational efficiency by decoupling runtime from dimensionality, instead scaling with the number of desired subtypes.

Dataset Name	Stages	Subtypes	Features	s-SuStaIn Wall Time	ReduXis Wall Time
TCGA-COAD	4	1	5092	1,350,368 s (15 d, 15 h)	224 s (3 m, 44 s)
TCGA-BLCA	5	1	5727	1,810,983 s (20 d, 23 h)	255 s (4 m, 15 s)
EHBS	4	3	71	1717 s (28 m, 47 s)	1393 s (23 m, 13 s)

**Table 4:** *Runtime complexity analysis of s-SuStaIn.* To characterize the scalability of the s-SuStaIn model, we empirically measured wall-clock runtime across varying numbers of subjects (M) while holding all other parameters constant within a simulated dataset (N = 800 patients) located within the validation folder of ReduXis (subtypes = 1, stages = M biomarkers, MCMC iterations during burn-in = 10,000 iterations, final MCMC iteration count = 100,000). Runtime increased nonlinearly with M, ranging from 31 s at M = 2 to 400 s at M = 100. For M > 100 biomarkers, the algorithm starts to spend more time resolving ambiguous biomarker orderings than performing likelihood evaluations.

Number of Biomarkers	s-SuStaIn Wall Time
2	30.76 s
3	33.43 s
5	36.79 s
10	47.15 s
25	71.74 s (1 m, 12 s)
50	132.44 s (2 m, 12 s)
100	398.50 s (6 m, 39 s)

**Table 5:** *Algorithmic estimation of the time complexity of s-SuStaIn.* We fit a curve, using a quasi-Newton algorithm called Limited-memory Broyden–Fletcher–Goldfarb–Shanno (L-BFGS) with Bound constraints and offered in the SciPy package in Python to runtime data using standard computational complexity models (e.g., O(logM), O(M), O(MlogM), $O(M^{2})$, $O(2^{M})$) based on the equation Time=k×f(M)+b, and evaluated each using root-mean-squared error (RMSE), which coincided as the objective function for the aforementioned gradient descent optimization method. Among all tested models, $O(M^{2})$ provided the best fit to the observed data (RMSE = 10.67 s), indicating that the algorithm scales quadratically with subject count. This is consistent with the design of the EM algorithm in s-SuStaIn, which includes a pairwise biomarker likelihood sorting step that inherently introduces $O(M^{2})$ complexity. These results highlight the importance of considering algorithmic scalability for large cohorts and motivate potential future optimizations.

Model	Fit Coefficient (*k*)	Base Coefficient (*b*)	RMSE (s)
O(logM)	50.081	0.0066	104.04
O(M)	3.597	7.06	27.47
O(MlogM)	0.539	21.47	18.90
$O(M^{2})$	0.036	30.02	10.65
$O(M^{3})$	0.000354	29.94	27.25
$O(2^{M})$	$1.14\times 10^{-50}$	$5.16\times 10^{-9}$	163.50

## Data Availability

Bulk RNA-seq cancer datasets, both of which are open access, were accessed from projects derived from The Cancer Genome Atlas (TCGA). For colorectal adenocarcinoma data, open-access data were obtained from the TCGA-COAD project, and for transitional cell carcinoma, from the TCGA-BLCA project. All datasets were accessed through the Genomic Data Commons (GDC) Data Portal https://portal.gdc.cancer.gov (accessed on 8 August 2025) under open-access conditions. Although an accession ID (phs000178.v11.p8, release date 18 December 2019) is associated with the broader TCGA dataset in dbGaP, no controlled-access data were requested, accessed, or used in this study, and no dbGaP approval or eRA Commons account was required. Data from the Emory Healthy Brain Study (EHBS) is available upon request by contacting James J. Lah at jlah@emory.edu. All code used in this study is available at the Laboratory for Pathology Dynamics GitHub repository under the MIT license: https://github.com/pathology-dynamics/ReduXis (accessed on 8 August 2025, version 1.0). Additional materials, including preprocessing scripts and tutorial images, are included in the repository and described in the Appendix A version-tagged release corresponding to this manuscript will be created upon acceptance, and the final release will include the DOI of the published paper in its documentation.

## Appendix A

This appendix presents Figures and tables that provide additional insights and support for the findings discussed in the main text.

**Table A1:** *Functional interpretation of top-ranked biomarkers selected by ReduXis in colorectal adenocarcinoma (CRAC).* This table summarizes the biological roles and mechanistic relevance of several high-importance features identified by ReduXis in the TCGA-COAD dataset. Each gene is annotated with its known molecular function and evidence-supported contribution to CRAC pathophysiology. Together, these biomarkers provide biological validation of the model’s inferred disease trajectory—from early disruptions in glycosylation and cell cycle regulation (e.g., *RPN2*, *KLF4*) to late-stage oncogenic activation and cytoskeletal remodeling (e.g., *TRIB3*, *GSN*). Literature citations are included for reference.

Gene	Functional Role	Mechanistic Relevance in CRAC
*TRIB3*	Scaffold pseudokinase; modulates AKT/mTOR pathway	Upregulated in CRAC; promotes tumor growth by enhancing β-catenin signaling and EMT. Drives proliferation, apoptosis evasion, and metabolic reprogramming [28].
*RPN2*	N-linked glycosylation; ER stress mediator	Acts as a proto-oncogene. Stabilizes glycosylated survival proteins and enhances chemoresistance (e.g., to oxaliplatin) by maintaining ER homeostasis [29].
*GSN*	Actin-severing protein; cytoskeletal regulation	Functions as a tumor suppressor. Downregulated in CRAC; inhibits invasion and proliferation via actin remodeling and STAT3 pathway suppression [30].
*KLF4*	Zinc-finger transcription factor; cell cycle arrest and differentiation	Tumor suppressor role. Downregulation is associated with poor survival and advanced grade; regulates p21 and p53-dependent DNA damage response [31].

**Table A2:** *Chi-squared goodness-of-fit analysis of stage distribution in colorectal cdenocarcinoma (TCGA-COAD).* This analysis evaluates whether clinical stage assignments deviate significantly from uniform random distributions across “Normal” and “Tumor” outcome groups within colorectal adenocarcinoma (N = 446). The data, obtained from the TCGA-COAD project under the TCGA program, were stratified by outcome and analyzed using $\chi^{2}$ goodness-of-fit tests. Substantial deviation was observed in both groups, with particularly strong skew in the tumor cohort, suggesting clinically meaningful stage-specific enrichment.

	Stage 1	Stage 2	Stage 3	Stage 4
**Subtype 1 (N = 446)**				
**Outcome: Normal (N = 39)**				
Observed:	14	16	7	2
Expected:	9.75	9.75	9.75	9.75
**Outcome: Tumor (N = 407)**				
Observed:	0	0	0	407
Expected:	101.75	101.75	101.75	101.75
$\chi^{2}$ = 1233.79, df = 3, *p* = 3.410 × 10^−267^ (***)				

**Global Chi-Squared Test (Null: Random Uniform Stage Assignment)**: $\chi^{2}$ = 1233.79, df = 3, *p* = 3.410 × 10^−267^ (***). *** *p* < 0.001.

**Table A3:** *Biological functions and oncogenic relevance of top-ranked biomarkers in transitional cell carcinoma (TCC).* This table summarizes select high-importance features identified by ReduXis in the TCC (TCGA-BLCA) cohort. Genes are categorized by their functional role—either as pro-tumorigenic effectors (e.g., *COL3A1*, *MXRA8*) or tumor suppressors (e.g., *IDH1*, *H1-2*)—and annotated based on literature-supported evidence of involvement in extracellular matrix remodeling, cell adhesion, chromatin regulation, or metabolic maintenance. These features contribute to the inferred staging trajectory of TCC progression and align with known mechanistic models of bladder cancer pathogenesis.

Gene	Functional Role	Mechanistic Relevance in TCC
*COL3A1*	Collagen III fibril; ECM structural integrity	Upregulated in TCC; promotes invasion and poor survival by activating integrin–FAK signaling and downstream PI3K/MAPK cascades, supporting ECM remodeling and metastasis [32].
*MXRA8*	ECM adhesion molecule; focal adhesion mediator	Overexpressed in advanced stages; implicated in EMT, cell motility, and metastatic potential via activation of FAK and cytoskeletal signaling [33].
*IDH1*	NADP^+^-dependent enzyme; redox and epigenetic homeostasis	Tumor suppressor; downregulation disrupts α-KG production, leading to hypermethylation and impaired DNA repair. Associated with increased genomic instability [34].
*H1-2*	Linker histone; chromatin compaction	Downregulated in aggressive TCC. Aberrant phosphorylation promotes chromatin relaxation and mitotic activity; linked to dysregulated gene expression and carcinogenesis [35].

**Table A4:** *Chi-squared goodness-of-fit analysis of stage distribution in transitional cell carcinoma (TCGA-BLCA).* This table presents results from a $\chi^{2}$ goodness-of-fit analysis evaluating whether clinical stage distributions deviate significantly from uniform random assignment across 3 outcome groups in transitional cell carcinoma (N = 343), based on data obtained from the TCGA-BLCA project under the TCGA program. The observed stage frequencies are compared against uniform expected counts (within each group) across 5 clinical stages. Strong deviation from randomness is evident, particularly within the Invasive and Metastatic subgroups. The global $\chi^{2}$ test confirms statistically significant outcome-stage structuring.

	Stage 1	Stage 2	Stage 3	Stage 4	Stage 5
**Subtype 1 (N = 343)**					
**Outcome: Localized (N = 19)**					
Observed:	8	3	3	4	1
Expected:	3.80	3.80	3.80	3.80	3.80
**Outcome: Invasive (N = 207)**					
Observed:	34	38	32	69	34
Expected:	41.40	41.40	41.40	41.40	41.40
**Outcome: Metastatic (N = 117)**					
Observed:	10	9	21	65	12
Expected:	23.40	23.40	23.40	23.40	23.40
$\chi^{2}$ = 126.80, df = 8, *p* = 1.300 × 10^−23^ (***).					

**Global Chi-Squared Test (Null: Random Uniform Stage Assignment)**: $\chi^{2}$ = 126.80, df = 8, *p* = 1.300 × 10^−23^ (***). *** *p* < 0.001.

**Table A5:** *Biological roles and pathological relevance of high-importance biomarkers in Alzheimer’s disease (AD).* This table summarizes select key biomarkers identified by ReduXis in the context of Alzheimer’s disease, with emphasis on their mechanistic roles in synaptic regulation, lipid homeostasis, neuroinflammation, and infection-mediated protein misregulation. Notably, several markers are influenced by oral infections, such as those caused by *P. gingivalis*, which has been implicated in the neuroinflammatory variant of AD.

Biomarker	Functional Role	Mechanistic Relevance in AD
*APOE*	Lipid transport; synaptogenesis; A β metabolism	Facilitates cholesterol transport and A β clearance. Dysfunctional apoE leads to impaired amyloid clearance and enhanced oxidative stress, contributing to plaque formation and neurodegeneration [36].
*DKK3*	Wnt signaling antagonist; synaptic regulation	Upregulated in early AD. Suppresses excitatory neurotransmission and promotes inhibitory tone. Localizes with A β plaques and drives network dysfunction; knockdown restores memory in mouse models [37].
*CST3*	Cysteine protease inhibitor; glial modulation	Highly upregulated in neuroinflammatory states. Amplifies cytokine signaling and glial activation. Overexpressed during infection-associated AD, particularly in cases involving *P. gingivalis*, which triggers reactive gliosis and blood–brain barrier breakdown [38].
*ALB*	Osmotic balance; A β sequestration; antioxidant	Decreased in AD. Normally binds and clears A β. *P. gingivalis* infection compromises the blood–brain barrier, allowing albumin to leak into the brain parenchyma, where it becomes pro-inflammatory and neurotoxic [39,40].

**Table A6:** *Chi-Squared Goodness-of-Fit Analysis of Stage Distributions in AD (EHBS Cohort, Inferred Subtypes).* This table presents results from a $\chi^{2}$ goodness-of-fit analysis evaluating whether observed distributions of disease stages (Stages 1–4) deviate from a uniform distribution across three clinical outcome groups (N = 392)—cognitively normal (Control), asymptomatic AD (AsymAD), and symptomatic AD (AD)—within each of three neurodegenerative subtypes inferred from the EHBS cohort using the ReduXis pipeline. The subtypes, derived from imaging and biomarker features, correspond to: Subtype 1 (non-inflammatory, amyloid-driven AD), Subtype 2 (inflammatory AD), and Subtype 3 (tau-driven cortical atrophy AD). For each outcome–subtype pairing, the null hypothesis assumes uniform random stage assignment, with no structured enrichment. To correct for multiple comparisons across the three subtype-specific tests within each clinical outcome group, Bonferroni correction was applied by multiplying the nominal *p*-values by 3. All corrected *p*-values remain statistically significant, indicating that stage distributions within outcome groups are non-random and subtype-specific. A global test across all 9 subtype–outcome combinations yields a highly significant result ($\chi^{2}$ = 382.31, df = 12, *p* = 2.096 × 10^−74^), providing strong evidence for structured stage stratification associated with both inferred subtype and clinical outcome.

	Stage 1	Stage 2	Stage 3	Stage 4
**Subtype 1 (N = 164)**				
**Outcome: Control (N = 53)**				
Observed:	1	11	41	0
Expected:	13.25	13.25	13.25	13.25
**Outcome: AD (N = 74)**				
Observed:	0	8	28	38
Expected:	18.50	18.50	18.50	18.50
**Outcome: AsymAD (N = 37)**				
Observed:	0	8	21	8
Expected:	9.25	9.25	9.25	9.25
$\chi^{2}$ = 157.48, df = 6, *p* = 6.066 × 10^−31^ (***)				
**Subtype 2 (N = 146)**				
**Outcome: Control (N = 57)**				
Observed:	0	16	38	3
Expected:	14.25	14.25	14.25	14.25
**Outcome: AD (N = 35)**				
Observed:	0	3	9	23
Expected:	8.75	8.75	8.75	8.75
**Outcome: AsymAD (N = 54)**				
Observed:	0	2	21	31
Expected:	13.50	13.50	13.50	13.50
$\chi^{2}$ = 148.82, df = 6, *p* = 4.122 × 10^−29^ (***)				
**Subtype 3 (N = 82)**				
**Outcome: Control (N = 23)**				
Observed:	0	6	7	10
Expected:	5.75	5.75	5.75	5.75
**Outcome: AD (N = 20)**				
Observed:	0	1	4	15
Expected:	5.00	5.00	5.00	5.00
**Outcome: AsymAD (N = 39)**				
Observed:	0	3	11	25
Expected:	9.75	9.75	9.75	9.75
$\chi^{2}$ = 76.01, df = 6, *p* = 7.134 × 10^−14^ (***)				

**Overall Chi-Squared Test (Null: Random Uniform Stage Assignment)**: $\chi^{2}$ = 382.31, df = 12, *p* = 2.096 × 10^−74^ (***). *** *p* < 0.001.
